# The role of fire in global forest loss dynamics

**DOI:** 10.1111/gcb.15591

**Published:** 2021-03-27

**Authors:** Dave van Wees, Guido R. van der Werf, James T. Randerson, Niels Andela, Yang Chen, Douglas C. Morton

**Affiliations:** ^1^ Department of Earth Sciences Vrije Universiteit Amsterdam Amsterdam The Netherlands; ^2^ Department of Earth System Science University of California Irvine CA USA; ^3^ School of Earth and Environmental Sciences Cardiff University Cardiff UK; ^4^ Biospheric Sciences Laboratory NASA Goddard Space Flight Center Greenbelt MD USA

**Keywords:** active fires, burned area, deforestation, fire, forest loss, satellite data, tree mortality

## Abstract

Fires, among other forms of natural and anthropogenic disturbance, play a central role in regulating the location, composition and biomass of forests. Understanding the role of fire in global forest loss is crucial in constraining land‐use change emissions and the global carbon cycle. We analysed the relationship between forest loss and fire at 500 m resolution based on satellite‐derived data for the 2003–2018 period. Satellite fire data included burned area and active fire detections, to best account for large and small fires, respectively. We found that, on average, 38 ± 9% (± range) of global forest loss was associated with fire, and this fraction remained relatively stable throughout the study period. However, the fraction of fire‐related forest loss varied substantially on a regional basis, and showed statistically significant trends in key tropical forest areas. Decreases in the fraction of fire‐related forest loss were found where deforestation peaked early in our study period, including the Amazon and Indonesia while increases were found for tropical forests in Africa. The inclusion of active fire detections accounted for 41%, on average, of the total fire‐related forest loss, with larger contributions in small clearings in interior tropical forests and human‐dominated landscapes. Comparison to higher‐resolution fire data with resolutions of 375 and 20 m indicated that commission errors due to coarse resolution fire data largely balanced out omission errors due to missed small fire detections for regional to continental‐scale estimates of fire‐related forest loss. Besides an improved understanding of forest dynamics, these findings may help to refine and separate fire‐related and non‐fire‐related land‐use change emissions in forested ecosystems.

## Graphical abstract

Fire is often a key process when considering the drivers of forest loss. Fire and forest loss can be directly related, such as in wildfires, but also indirectly because of slash burning after tree felling or tree mortality after fire damage. Using a novel approach for combining satellite‐derived forest loss with fire data from burned area and active fire detections, we found that 38 ± 9% of the global forest loss during 2003–2018 was fire‐related. We discuss global patterns and trends in fire‐related forest loss, and compare our results for fire data of different spatial resolutions.

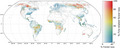

## 1 INTRODUCTION

Forests play a crucial role in the climate system and are integrally linked to biodiversity, biogeochemical and hydrological cycling, and the Earth's radiation budget (Foley et al., 2005). Global estimates of gross forest loss during 2001 to 2018 totalled 4.2 million km^2^ (Hansen et al., 2013). Large‐scale forest loss can significantly reduce the land carbon sink, modify the surface energy budget and affect cloud formation, with impacts on regional weather and global climate (Bonan, 2008; Pongratz et al., 2011; Swann et al., 2018). When forest loss is followed by recovery and regrowth, these biogeochemical and biogeophysical impacts can be partly or fully offset over time. This is not the case if the forest loss is permanent as a consequence of land use change, climate or other global change drivers.

Fire is often a key process when considering the drivers of forest loss. At least half of the global forest loss may be caused by a combination of natural and anthropogenic drivers that are in principle associated with fire, such as wildfire, or that involve the use of fire for clearing or slash burning, such as with commodity‐driven deforestation or shifting agriculture (Curtis et al., 2018). In regions where wildfires are dominant, including the boreal forests of North America and Eurasia, forest dynamics and fire are closely linked (Kasischke et al., 2002; Krylov et al., 2014; Wang et al., 2021). Compared to the boreal region, where ignition by lightning plays a crucial role (Veraverbeke et al., 2017), wildfires in temperate regions are often closer to human settlements and more likely to be ignited, but also suppressed, by humans (Balch et al., 2017).

Besides forest loss from wildfire, fire is widely used by humans as an inexpensive and effective tool for managing and transforming land for agriculture. Commodity‐driven deforestation for the development of pasture or cropland is the main driver of permanent forest loss in the humid tropics (Kim et al., 2015; Morton et al., 2008). This process typically involves mechanical felling of all standing vegetation, followed by repeated burning of the resulting slash (Carvalho et al., 1995; Kauffman et al., 1995). These activities may lead to substantial greenhouse gas emissions, potentially with considerable additional emissions due to fires escaping into neighbouring forests (Aragão et al., 2018; Siegert et al., 2001; Tyukavina et al., 2018) or into peat soils (Page & Hooijer, 2016; Turetsky et al., 2015), especially during drought periods.

Shifting agriculture, commonly practised in sub‐Saharan Africa, and parts of Middle and South America and Equatorial Asia (Curtis et al., 2018), typically involves much smaller clearings and short cycles of forest loss, land use and regrowth (Molinario et al., 2017; Potapov et al., 2012). The land conversion generally involves the use of fire for land clearing and soil regeneration, that is, ‘slash‐and‐burn’. In parts of Sub‐Saharan Africa, this type of forest loss is becoming increasingly permanent as a consequence of agricultural expansion, shorter fallow times and demand for charcoal, often linked to increasing population pressure (Ickowitz et al., 2015; Sedano et al., 2020; Tyukavina et al., 2018).

Disturbance agents other than fire that can lead to forest loss include drought, wind storms, insect and disease outbreaks (Goulden & Bales, 2019; Kurz et al., 2008), and human activities such as logging, forest clearing for mining activities or urban expansion (Asner et al., 2013). Forests degraded by these disturbance agents may become more vulnerable to subsequent drought and fire (Cochrane et al., 1999; Kurz et al., 2008; Siegert et al., 2001). Climate change is likely to alter forests worldwide (Bonan, 2008), influencing many aspects of forestry (Kirilenko & Sedjo, 2007), and increasing the frequency and intensity of some or all of the natural drivers of forest loss (Pugh et al., 2019).

Satellite data records show that forest loss rates have varied substantially over the past several decades, with pronounced shifts in tropical deforestation dominating the global trend. Tropical forest loss increased since about 1990 (Kim et al., 2015) and reached a maximum around 2004 due to peak levels of deforestation in the Brazilian Amazon (Turubanova et al., 2018). The subsequent decrease in tropical forest loss in the Brazilian Amazon until 2010 as a result of, for example, efforts to expand protected areas and prevent illegal deforestation (Gibbs et al., 2015; Koren et al., 2007; Nepstad et al., 2009), was offset by an increase in other parts of South America and ongoing increases in Indonesia and Africa (Hansen et al., 2013; Kim et al., 2015; Margono et al., 2014; Turubanova et al., 2018; van Marle et al., 2017). In recent years, tropical forest loss rates in Indonesia have declined substantially (Carlson et al., 2018; Gaveau et al., 2019). A recent study by Song et al. (2018) showed that global forest loss from 1982 to 2016 has not resulted in a net decline in global forest cover, as the decline in tropical forest cover has been offset by forest gains in the extratropics.

Although the role of fire in forest loss has been evaluated on regional and global scales using a variety of different satellite sensors, estimates remain uncertain. In particular, global studies typically rely on moderate resolution burned area data that omit smaller fires that are more likely to be captured by active fire detections (Achard et al., 2014; Baccini et al., 2017; Liu et al., 2019; Pearson et al., 2017; van der Werf et al., 2017). For example, Liu et al. (2019) found that only 15% of global forest loss was fire‐related. Their approach relied on Landsat‐based forest loss data in combination with MODIS‐based burned area for regions with at least 20% tree cover, excluding the role of small fires. An improved understanding of the role of fire in global forest loss is a critical step towards estimating land‐use change emissions and predicting the future role of forests in the global carbon cycle.

Here we aim to improve this understanding using a novel approach for estimating the fraction of gross (permanent and non‐permanent) forest loss that involves fire on a global scale, which we refer to as fire‐related forest loss. In this estimate, we include forest loss that is the direct result of fire, such as in wildfires, but also indirect fire‐use during land conversion practices such as slash burning as part of commodity‐driven deforestation and shifting agriculture. In contrast to previous studies by Curtis et al. (2018) and Tyukavina et al. (2018), we focus on quantifying the fraction of forest loss that is related to fire, regardless of what driving mechanism is at play. Besides accounting for small fires by incorporating active fire detections based on a statistical method, our approach also allows for a more detailed analysis of the spatial and temporal dynamics in both forest loss and fire. We combine Landsat‐derived forest loss data (Hansen et al., 2013) with Moderate resolution Imaging Spectroradiometer (MODIS) burned area (Giglio et al., 2018) and active fire detections (Giglio et al., 2016) on a 500 m resolution global grid. We examine overlap between forest loss and fire detections both spatially and temporally to better understand the data constraints that limit the accuracy of fire‐related forest loss estimates. Our analysis also considers differences in the timing and uncertainty of change detection among the fire and forest loss products, spanning daily (active fire detections) to annual (forest loss) time‐scales. We compare our approach based on MODIS active fires to higher‐resolution estimates based on active fire detections from the Visible Infrared Imaging Radiometer Suite (VIIRS; Schroeder et al., 2014) and 20 m resolution burned area from the Sentinel‐2 Multispectral Instrument (MSI; Roteta et al., 2019), to test the validity of the coarser‐resolution approach.

## 2 METHODS

### 2.1 Datasets

Global fire‐related forest loss was estimated for each year during the 2003–2018 period by overlaying 500 m gridded fractional forest loss with burned area, supplemented by active fires outside the perimeter of burned area pixels. Annual forest loss for the period 2001–2018 was based on the Global Forest Change (GFC) version 1.6 dataset (Hansen et al., 2013), which was derived from Landsat 7 Enhanced Thematic Mapper Plus (ETM+) and Landsat 8 Operational Land Imager (OLI) satellite data at 30 m resolution. Data inconsistencies in the forest loss dataset, including the transition from Landsat 7 to 8, are discussed in Data S1. We aggregated the annual forest loss data to the MODIS 500 m sinusoidal grid with units of fractional forest loss area per 500 m pixel. We used the 2000–2012 single time step ‘forest gain’ layer from the GFC dataset as a reference for the location of regions with forestry activity or tree plantations (Hansen et al., 2013). In this data layer, forest gain is defined as the inverse of forest loss. The distribution of fire‐related forest loss over tree cover intervals was analysed using the Landsat‐derived 30 m fraction tree cover layer for the year 2000 from the GFC dataset (as in e.g. Baccini et al., 2017; Carlson et al., 2018; Gaveau et al., 2019; Hansen et al., 2013; Zarin et al., 2016). Besides tree cover intervals, we also analysed fire‐related loss of primary humid tropical forests using the 30 m resolution primary humid tropical forest mask for the year 2001 produced by Turubanova et al. (2018).

Fires were identified using both burned area and active fire detections. Our main estimate was based on 500 m resolution Aqua and Terra MODIS MCD64A1 Collection 6 burned area (Giglio et al., 2018) in combination with MODIS MCD14ML Collection 6 active fire locations (1 km at nadir; Giglio et al., 2016). For comparison, we also combined the MODIS burned area with higher‐resolution Suomi National Polar‐orbiting Partnership (NPP) VIIRS I‐band VNP14IMGML active fires (375 m at nadir; Schroeder et al., 2014). MODIS data were available for the whole study period, whereas VIIRS data were only available from 2012 onwards. Besides VIIRS, we made a second assessment using 20 m resolution FireCCISFD11 burned area for sub‐Saharan Africa for the year 2016 based on Sentinel‐2 MSI (Roteta et al., 2019).

### 2.2 Gridding of fire location products

Fire locations from the MODIS and VIIRS active fire products were first converted to active fire pixels (i.e. orbital swath pixels; Giglio et al., 2016), by projection onto the Earth's surface based on the satellite scan geometry, and then in a second step reprojected onto the 500 m MODIS sinusoidal grid. Once projected, one MODIS active fire pixel can span at least four 500 m pixels at nadir and up to 40 at the scan edge, depending on the scan angle and the associated along‐scan and along‐track pixel dimensions. The same approach was used for gridding of the VIIRS active fire locations to the 500 m grid, but using the VIIRS‐specific scan geometry.

Besides the higher nadir resolution of the VIIRS sensor compared to MODIS, off‐nadir pixel growth is strongly reduced for VIIRS due to a pixel aggregation method. This results in a pixel area of 0.14 km^2^ at nadir and up to 0.62 km^2^ at the scan edge for VIIRS (up to two 500 m pixels), compared to about 1 km^2^ at nadir and up to 10 km^2^ at the scan edge for MODIS (up to 40 500 m pixels), which is a factor 7 to 16 larger in area (Schroeder et al., 2014). The smaller active fire pixel size for VIIRS leads to a higher detection probability because of larger contrast of the thermal anomaly compared to the radiometric background (Csiszar et al., 2014). Furthermore, a larger swath width (3060 km) ensures the overlap of consecutive swaths and full global coverage every 12 h. In comparison, the two MODIS sensors combined have an overpass frequency of four times every 24 h, but the narrower swath (2330 km) leaves part of the tropics undetected for each specific day.

### 2.3 Determination of fire‐related forest loss

Fire‐related forest loss for 2003–2018 was defined as forest loss overlapping with fire detections (burned area or active fires), after reprojection of all required data onto the global 500 m MODIS sinusoidal grid (Figure S1). Our definition of fire‐related forest loss included any sequence of causality between fire and forest loss, including simultaneous occurrence of fire and forest loss (e.g. wildfire), fire followed by forest loss (e.g. tree mortality after fire damage) and forest loss followed by fire (e.g. burning of slash after felling, which mostly happens in the same year as the felling).

In our fire‐related forest loss estimate, we included forest loss pixels overlapping with fire detections from the current year (t=0) and the first preceding year (t=‐1). This decision was based on an exploratory lag analysis in which we looked at the overlap of monthly fire detections in a range from 2 years preceding to 2 years succeeding the forest loss detection year (t=‐2tot=+2; Figure S2). The first preceding year accounted for one‐third of fire‐related forest loss globally and up to about half in the boreal region (Figure S2, percentages displayed above lag years), which showed that a substantial part of the forest loss detections was delayed, likely as a consequence of post‐fire tree mortality and temporal differences among the forest loss and fire data products. This has been accounted for in previous studies by using a multi‐year pre‐forest loss fire buffer (Krylov et al., 2014; Liu et al., 2019). In our study, a 1‐year buffer based on the first preceding year proved to be sufficient to include the majority of overlap between forest loss and fire detections while minimizing commission errors (i.e. fire unrelated to forest loss; Figure S2).

The 1‐year buffer also accounted for fire seasons that occurred at the transition of two calendar years, such as the northern African fire season lasting from December to February. MODIS burned area and active fire detections for 2002–2018 were used to calculate fire‐related forest loss for the 2003–2018 period (minus 2002, the first preceding year). Similarly, VIIRS active fire detections became available in 2012 and were separately used to calculate fire‐related forest loss for 2013–2018. Although the fire‐related forest loss estimate for 2003 was incomplete because of the MODIS Aqua satellite record starting in May 2002, the majority of the global fire year of 2002 was captured and the additional fire‐related forest loss in the first 4 months was minor (see Figure S2, months −12 to −8).

All forest loss overlapping with burned area in the forest loss detection year and the preceding year was considered to be directly or indirectly related to fire. We used the total forest loss area within a burned area pixel, ${\text{FL}}_{\text{tot}}$, as the mean estimate of the fire‐related forest loss area based on burned area, ${\text{FL}}_{\text{burned area}}$:

(1) $${\text{FL}}_{\text{burned}\;\text{area}}\left(i,j\right)={\text{FL}}_{\text{tot}}\left(i,j\right)\pm R_{\text{burned}\;\text{area}}\left(i,j\right).$$

The uncertainty range, $R_{\text{burned}\;\text{area}}$, is defined as the range in estimates found as a result of three different adjustments to the algorithm based on the approach by Liu et al. (2019), namely, (1) inclusion of lag year t=‐2, (2) exclusion of 500 m pixels with more than one burned area detection in a single year and (3) exclusion of forest loss in 30 m pixels with less than 20% fraction tree cover (Figure S3).

Three main factors motivated our approach of assigning all forest loss overlapping with burned area as being fire‐related. First, for a confident burned area detection at least half, and often more, of a 500 m pixel $\left(i,j\right)$ has to be burned with considerable burn intensity (Giglio et al., 2009). Second, the annual average fraction of forest loss in a 500 m pixel was substantially higher for pixels overlapped by burned area, as compared to those without overlap (compare Figure S4a,b). This shows that, where burned area and forest loss overlapped, forest loss events were relatively large scale, suggesting a large likelihood of fire‐relatedness. Third, homogeneous regions where forest loss overlapped with fire detections were often clearly spatially separated from regions without overlap. Furthermore, whether forest loss was mostly overlapped by burned area or active fire detections was also region‐specific, indicating characteristic fire regimes and forest loss drivers. For large parts of Africa, for example, we found a strong spatial separation between large fires and forest loss, discriminating large savanna surface fires that leave trees unaffected (often detected as burned area) from small‐scale shifting agriculture fires related to forest loss (predominantly detected as active fires; Figure S1a,b).

In 500 m pixels without burned area, we used active fire detections to identify fire‐related forest loss. Active fire detection methods are better able to capture small fires, but this is limited to satellite overpasses without cloud obstruction. Fires detected in the 1 km MODIS active fire product can be up to a factor of about 1000 smaller than the minimum detectable size of a burn scar, as long as the fire radiative power is sufficient (Giglio et al., 2006). The spatial relationship between forest loss and active fire detections is therefore less straight‐forward than with burned area detections, for two reasons. First, an active fire pixel can be substantially larger than the forest loss event, especially if the fire detection is off‐nadir (due to the scan angle effect). Second, the location, size and number of individual fires inside an active fire pixel are unknown and the actual fire perimeter can be substantially smaller than the active fire pixel footprint.

To account for the relatively uncertain relationship between active fire detections and forest loss, we calculated lower‐ and upper‐bound probability measures ($P_{\text{min}}$ and $P_{\text{max}}$) based on the spatial overlap between forest loss and active fire pixels. A schematic example for the calculation of active fire‐based fire‐related forest loss is shown in Figure S5. We estimated the relatedness of all active fire detections in the current and preceding year coinciding with forest loss for every 500 m pixel with indices $\left(i,j\right)$. Forest loss in a 500 m pixel was given a probability of being fire‐related if the pixel midpoint was located within the perimeter of the swath‐based active fire footprint. The probability that a single overlapping active fire detection was related to the forest loss was calculated based on the ratio between the area of forest loss overlapping the active fire pixel and the total area of that active fire pixel, multiplied by the active fire detection confidence:

(2) $$P_{\text{single}\;\text{fire}}=\frac{\sum_{f}A_{\text{forest}\;\text{loss}}\left(i,j\right)}{A_{f}\left(i,j\right)}C_{f}\left(i,j\right),$$

where $A_{f}$ is the fire pixel area (often multiple 500 m pixels) associated with a single active fire detection, $\sum_{f}A_{\text{forest}\;\text{loss}}$ is the total area of forest loss overlapping the active fire pixel and $C_{f}$ is the active fire detection confidence. For MODIS, the detection confidence, $C_{f}$, is given in percentage from 0% to 100%, whereas for VIIRS qualitative classes of ‘low’, ‘nominal’ and ‘high’ are provided, representing 15%, 55% and 90% confidence, respectively. In Equation (2), we divide these percentages by 100 for $C_{f}$ to range from 0 to 1. In turn, the probability that from all overlapping active fire pixels in the current and preceding year at least one (i.e. $P\left((\geq 1\right)$) detection was related to forest loss was calculated as:

(3) $$P_{\min}\left(\text{fire}\;\text{forest}\;\text{loss}\right)\left(i,j\right)=1‐\prod_{f=1}^{\#\;\text{fires}\;\text{in}\;\text{year}\;t=0\;\text{and}\;t=‐1}\left(1‐\frac{\sum_{f}A_{\text{forest}\;\text{loss}}\left(i,j\right)}{A_{f}}C_{f}\right),$$

which we defined as the minimum probability of fire‐relatedness, $P_{\text{min}}$.

The probability $P_{\text{min}}$ increases with every additional overlapping active fire pixel, representing that the detection of multiple active fires increases the likelihood that fire activity was indeed related to forest loss. Our method is purely based on an increase in probability with repeated fire detections and does not require empirical thresholds for detection confidence, number of detections or scan angle. In contrast, previous studies used, for example, a fire persistence threshold to distinguish smallholder agricultural burning from large‐scale deforestation (Morton et al., 2008).

For the second probability measure, active fires were treated the same as burned area, that is, it was assumed that the gridded active fire pixels were completely burned with a detection confidence of a 100%. In this case, the probability is always 1:

(4) $$P_{\max}\left(\text{fire}\;\text{forest}\;\text{loss}\right)\left(i,j\right)=1.$$

This upper level probability must be regarded as the maximum estimate for the available active fire detections and could overestimate fire‐related forest loss for those detections in cases where forest loss occurs in only a small fraction of a 500 m pixel.

Following the comparison of our MODIS‐based estimate to the higher‐resolution VIIRS and Sentinel‐2 ‐based estimates (see below), we concluded that the average of $P_{\min}$ and $P_{\max}$ provides the best estimate of fire‐related forest loss derived from active fires. The range between the two probabilities functions as a measure for the uncertainty that arises from the resolution mismatch between the forest loss and active fire data. Multiplication of the average probability with the forest loss area in the respective 500 m pixel gives the estimated area of forest loss related to active fires:

(5) $${\text{FL}}_{\text{active}\;\text{fire}}\left(i,j\right)=\left(\frac{P_{\min}\left(i,j\right)+P_{\max}\left(i,j\right)}{2}\pm \frac{P_{\max}\left(i,j\right)‐P_{\min}\left(i,j\right)}{2}\right){\text{FL}}_{\text{tot}}\left(i,j\right),$$

where ${\text{FL}}_{\text{active}\;\text{fire}}$ is the estimated fire‐related forest loss area derived from active fires. Considerations with regard to the sensitivity of ${\text{FL}}_{\text{active}\;\text{fire}}$ to the scan geometry are discussed in Data S1 and associated Figures S6 and S7. The final estimate, ${\text{FL}}_{\text{fire}}$, combines the burned area and active fires components. Active fires complement the estimate for 500 m pixels where burned area detections are not available:

(6) $${\text{FL}}_{\text{fire}}\left(i,j\right)={\text{FL}}_{\text{burned area}}\left(i,j\right)+{\text{FL}}_{\text{active}\;\text{fire}\;\notin \text{burned}\;\text{area}}\left(i,j\right).$$

Active fires are considered in any 500 m pixel without burned area, also when an active fire orbital swath pixel partially overlaps with burned area. The VIIRS‐based estimate also used MODIS burned area, but in combination with active fires from VIIRS instead of MODIS.

Fire‐related forest loss using FireCCISFD11 20 m Sentinel‐2 burned area was calculated on the 30 m resolution grid native to the GFC dataset, to fully exploit the correspondence in spatial resolution. A 30 m forest loss pixel was considered fire‐related if at least 30% of the pixel overlapped with 20 m Sentinel‐2 burned area. The sensitivity of the chosen overlap threshold on the resulting fire‐related forest loss estimate was determined by calculating additional minimum and maximum estimates based on pixel overlap thresholds of 50% and 10%, respectively. The range between the minimum and maximum estimates served as an uncertainty range. Fire‐related forest loss was calculated for 2016 Sentinel‐2 burned area in relation to forest loss for the years 2014–2018 to analyse the causality between products, similar to the lag analysis performed for the MODIS and VIIRS fire data described earlier, but by lagging forest loss instead of fire detections.

### 2.4 Data comparison and trend calculations

After determination of fire‐related forest loss at 500 m resolution, and 30 m for the Sentinel‐based approach, all results were aggregated to a 0.25° global grid for further analysis. MODIS‐based fire‐related forest loss was compared to the higher‐resolution VIIRS and Sentinel‐2 estimates by calculation of omission and commission errors at 500 m resolution and summation of these errors over larger regions (Table S1). Trends in the fraction of fire‐related forest loss, burned area and active fire detections were determined at an annual time step (2003–2018) using the non‐parametric Mann–Kendall test and Sen's slope estimator for the robustness of trends. We limited our trend analysis to the fraction of fire‐related forest loss, instead of absolute forest loss areas, to reduce the sensitivity of our analysis to inconsistencies in the GFC dataset time series (see Data S1). All trend calculations were performed using MODIS burned area and active fire detections, whereas VIIRS active fires were only used for comparison because VIIRS data were only available for 2012–2018. Trend maps for all significance levels are shown because non‐significant trends were often found to be part of spatially homogeneous patterns that conveyed information about larger‐scale regional trends. The fraction of interannual variability in forest loss explained by fire‐related forest loss was calculated as the squared Pearson's correlation coefficient.

## 3 RESULTS

### 3.1 Global patterns

**FIGURE 1:**
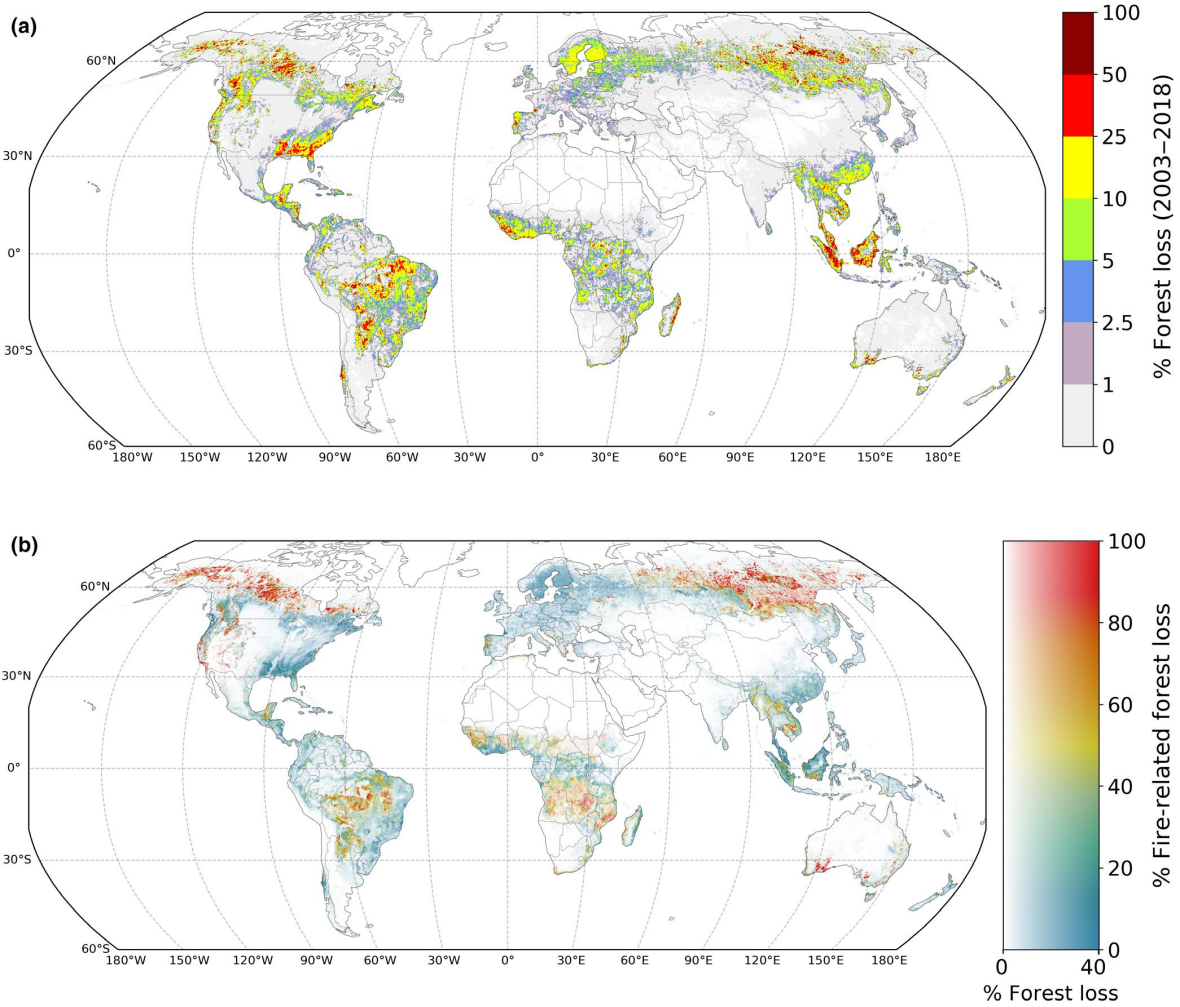
Global forest loss and fire‐relatedness, (a) percentage of 0.25° grid cell that underwent forest loss during 2003–2018 and (b) best‐estimate fraction of fire‐related forest loss. In panel (b), 0.25° grid cells that underwent less than 0.1% forest loss are masked out. The horizontal dimension of the colour map in panel (b) represents the percentage of forest loss from panel (a), scaled to the power of 0.5 and clipped at 40% forest loss for improved visualization

During 2003–2018, the average annual forest loss was 239 ⋅10^3^ km^2^ year^−1^ (Hansen et al., 2013), of which 38 ± 9% (91 ± 22 ⋅10^3^ km^2^ year^−1^) was fire‐related (Figure 1; Table 1). In sparsely populated parts of the boreal region, almost all forest loss was fire‐related, whereas in regions dominated by forestry, such as the Southeastern United States, Scandinavia and parts of China, the fire‐related fraction was typically 10% or less. In the tropics and subtropics, this fraction varied more and showed characteristic patterns consistent with drivers of forest loss (Figure S1). Fire‐related forest loss contributed to 89 ± 5% of the interannual variability in global forest loss rates, highlighting that the annual variation in forest loss was predominantly explained by drivers involving fire.

**TABLE 1:**
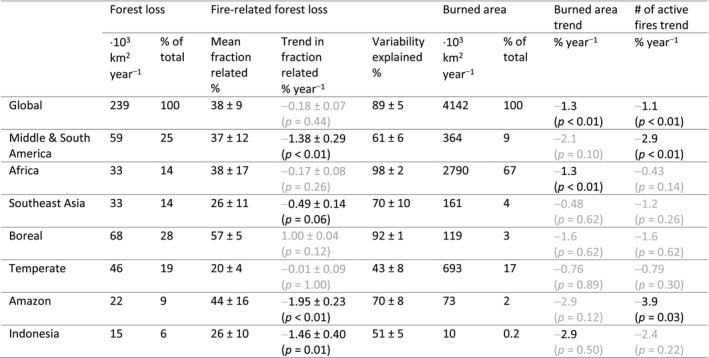
Annual 2003–2018 averages and trends of forest loss, fire‐related forest loss, burned area and active fire detections for different regions of the world. The column ‘Variability explained’ shows the fraction of interannual variability in forest loss that is explained by fire‐related forest loss (see Section 2). Annual averages and trends in burned area and number of active fires are shown for all fire detections (in‐ and outside forests). Continental regions are based on grouped GFED regions (see figure 3 in van der Werf et al., 2017): Middle and South America (CEAM, NHSA and SHSA), Africa (NHAF and SHAF), Southeast Asia (SEAS and EQAS), Boreal (BONA and BOAS) and Temperate (TENA, EURO, CEAS and AUST). Light grey coloured numbers indicate values that are not significant based on the Mann–Kendall test (*p* > 0.05). Forest loss areas based on Hansen et al. (2013)

**FIGURE 2:**
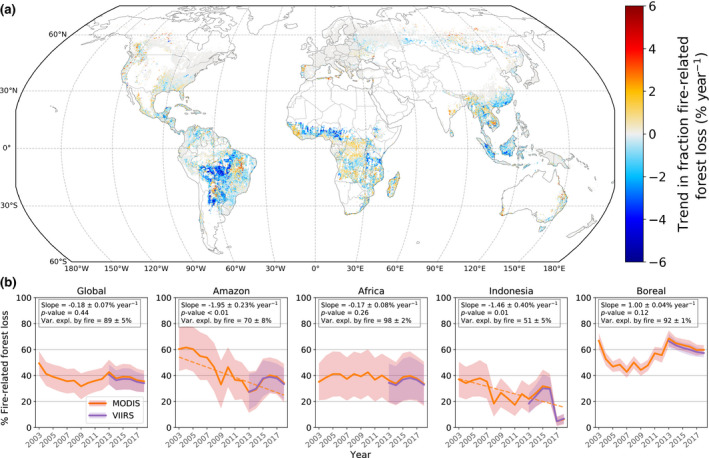
(a) Global trends in the fraction of fire‐related forest loss (2003–2018) per 0.25° grid cell based on MODIS burned area and active fires. (b) Annual fraction of fire‐related forest loss for key regions (see also Table 1), including the estimate based on VIIRS instead of MODIS active fires for 2013–2018. Transparent filled bands display the range between minimum‐ and maximum‐estimate fire‐related forest loss. Slope values and their significance, and the fraction of interannual variability in forest loss explained by fire‐related forest loss (Var. expl. by fire) are given per region. Only significant trend lines are plotted

The global average fraction of fire‐related forest loss remained stable over the study period. However, regional trends were significant, with marked declines in regions that were deforestation frontiers early in our study period (e.g. Amazon, Indonesia) and increases in the tropical forests of Africa (Figure 2). Local trends in regions with large interannual variability and (multi‐) decadal fire‐return intervals, such as the boreal region and Western North America, were often insignificant based on the Mann–Kendall test, although they appeared significant based on simple linear regression. In these regions, individual years with severe fire and large forest losses heavily influence trends, and therefore a longer time period is required to find robust trends. Trends in the fraction of fire‐related forest loss generally coincided well with trends in burned area and active fire detections (Figure S8).

Of the global fire‐related forest loss estimate, 23 ± 2% was captured by burned area and 15 ± 9% was added by active fires (Figures S7f and S9). Globally, 6.9% of burned area detections and 15.4% of active fire detections overlapped with forest loss (Figure S10a,b), indicating that only a small fraction of total fire detections was related to forest loss. The relative contribution of active fire detections to the total fire‐related forest loss estimate was largest in interior tropical forests and heavily managed temperate forests (Figure S10c). In temperate forests however, the amount of fire‐related forest loss was generally very minor.

### 3.2 Tropics

**FIGURE 3:**
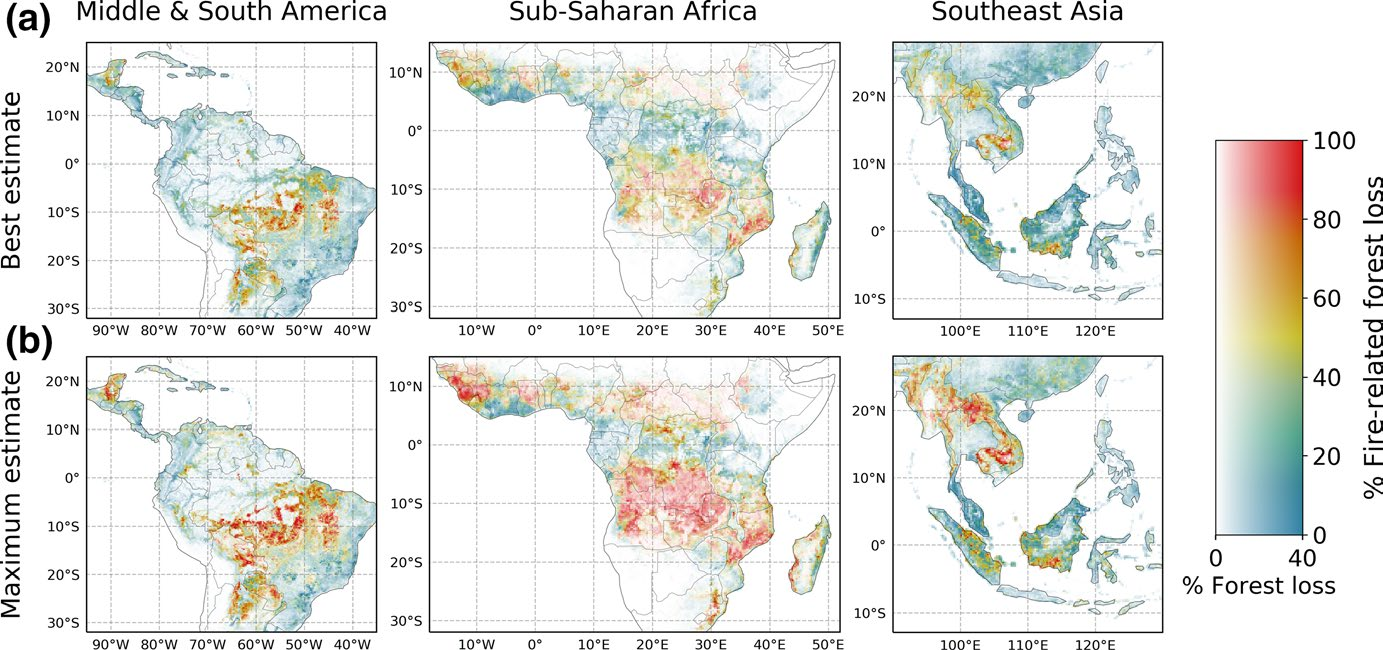
Comparison of 2003–2018 average (a) best‐estimate fire‐related forest loss and (b) maximum‐estimate fire‐related forest loss, for the regions Middle and South America, sub‐Saharan Africa and Southeast Asia. The difference between best and maximum estimates was largest for these three regions, whereas it was negligible for most temperate and boreal regions. The horizontal dimension of the colour map represents the percentage of forest loss from Figure 1a, scaled to the power of 0.5 and clipped at 40% forest loss for improved visualization, identical to the colour map of Figure 1b

**FIGURE 4:**
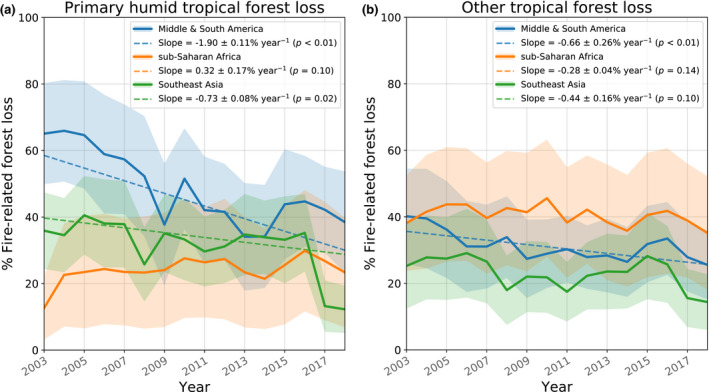
Annual fraction of fire‐related forest loss in the tropics (23.5°N–23.5°S) for (a) primary humid tropical forest and (b) other forest loss in the tropics. Region definitions as in Table 1, but limited in latitude to 23.5°N–23.5°S. Transparent filled bands display the range between minimum‐ and maximum‐estimate fire‐related forest loss. Only significant trend lines are plotted

Of all forest loss in the tropics (48% of global forest loss; 23.5°N–23.5°S), 34 ± 14% was fire‐related (Table 1; Figures 1 and 3). For primary humid tropical forests, the fraction of fire‐related forest loss was higher (41 ± 14%), of which 69% occurred in the tropical Americas, 22% in Southeast Asia, and only 8% in sub‐Saharan Africa (Figure 4). Compared to the tropical Americas and Asia, the forest loss regime in Africa consisted of more small‐scale forest loss, with more than three‐quarters of all forest loss area occurring in events <5% the size of a 500 m pixel (Figure S4a). For Africa, the majority of fire activity and forest loss occurred in the 25%–75% fraction tree cover range, whereas only 20% of fire‐related forest loss area occurred in the >75% tree cover range, compared to 68% for South America (Figure S11).

In the Amazon, fire‐related forest loss accounted for 44 ± 16% of the region's total forest loss and this drove interannual variability. Burned area detections accounted for about half of the fire‐related forest loss estimate in the Arc of Deforestation, an area dominated by large‐scale commodity‐driven deforestation, whereas the relative importance of active fire detections increased towards the rainforest interior (Figure S10c). The Amazon biome experienced the strongest decrease in the fraction of fire‐related forest loss of any region, declining from a mean level of 66 ± 16% in 2003–2005 to a mean level of 43 ± 15% in 2016–2018 (Figure 2).

In Africa, fire‐related forest loss amounted to 38 ± 17% of total forest loss, with a major role in large areas of Western and Southeastern Africa, and a less significant role in most African humid tropical forests (Figures 1 and 3; Figure S1a,b). The fraction of fire‐related forest loss remained stable during the study period for the African continent as a whole, whereas local trends showed a distinct pattern (Figure 2). Negative trends were found for northern and eastern Africa in areas where the fire‐related forest loss estimate was predominantly based on burned area detections, whereas positive trends were found for the tropical forest regions of Africa where burning was more frequently detected as active fires. While burned area decreased in most regions of Africa (−1.3% year^−1^; Table 1), active fire detections increased across much of the continent, particularly where burned area levels were low, such as in the tropical forest regions (Figure S8).

In Southeast Asia, fire‐related forest loss was 26 ± 11% (Figure S1c). The relative contribution of active fire detections compared to burned area detections was particularly large for this region (72% on average, Figure S10c). A difference in forest loss regimes was visible between parts of maritime Southeast Asia compared to the mainland (Indo‐Burma region). The maritime region, and in particular the Indonesian provinces of Riau (Sumatra) and Central Kalimantan, has seen extensive historical deforestation and conversion to agricultural land, mostly for palm oil (Figure 1; Austin et al., 2017; Gaveau et al., 2019). Compared to maritime Southeast Asia, forest loss in the mainland occurred in smaller patches, suggesting a larger role by smallholders (Figure S4a,b). Forest loss in Laos and Cambodia was predominantly related to fire, whereas fire played a smaller role in Vietnam, where policy changes favoured reforestation and logging (Meyfroidt & Lambin, 2008).

### 3.3 Boreal and temperate forest regions

In the boreal region, 57 ± 5% of forest loss was fire‐related and this explained 92 ± 1% of the interannual variability in forest loss (Table 1; Figure S1d). In remote northern areas, the share of fire‐related forest loss was often close to 100% as a consequence of large stand‐replacing wildfires, whereas in more productive forestlands further south, forest loss was a mixture of fire and forestry (Figure 1b). In boreal North America, these two forest loss regimes were clearly separated, whereas the transition was more gradual in boreal Asia, indicating a mixture of wildfires, forestry and agricultural burning in closer proximity. Our fire‐related forest loss estimate for the boreal region was particularly robust because much of the fire activity was detected as burned area (Figures S9 and S10) and because there was a strong spatial overlap between forest loss and large wildfires (Figure S1d).

In temperate regions, forest loss was dominated by forestry and fire played a minor role, except for wildfire‐prone areas such as California, Portugal, Greece, Australia and temperate Russia. Where major wildfires occurred, the interannual variability in forest loss was largely determined by these events. In areas with managed forests, including many regions in the Southeastern United States and China for example, the small amount of fire‐related forest loss found was almost fully based on active fire detections, indicating small‐scale human fire‐use (Figure S10c).

### 3.4 Estimates from higher‐resolution fire data

Fire‐related forest loss estimates for 2013–2018 based on MODIS and VIIRS active fires were similar, with 38 ± 9% and 37 ± 8%, respectively (slope = 1.03, *R* ^2^ = 0.99) and a 17% reduction in uncertainty for VIIRS (Figure 2b; Figure S12). Minimum estimates were particularly similar and differed by less than 1% globally, with minor opposing omission and commission errors (OE = 9%, CE = 9% at a global scale; Table S1). For the maximum estimate, commission errors played a larger role (OE = 15%, CE = 22%), leading to a 3% higher global estimate for MODIS (47% vs. 44% fire‐related). The average MODIS active fire pixel size weighted by forest loss area was 2.7 ± 1.0 km^2^ (±standard deviation), versus 0.25 ± 0.05 km^2^ for VIIRS, which translates to an average pixel size ratio for detections overlapping forest loss of 11 on average, with a standard deviation of 4.5 (Figure S13). Although the nadir resolution differed by a factor of 7 (1 km^2^ for MODIS versus 0.14 km^2^ for VIIRS), the overall resolution difference was larger due to the reduced off‐nadir pixel growth for VIIRS and because of an above‐average pixel size ratio in forest loss areas.

The fire‐related forest loss estimate for 2016 sub‐Saharan Africa based on the FireCCISFD11 Sentinel‐2 MSI product was 38 ± 3% and thus higher than the estimates based on MODIS (29 ± 14%) and VIIRS (28 ± 12%), but within their uncertainty range (Figure S14). For lagged forest loss years (2014–2018), the Sentinel‐2 estimate was more equal and sometimes slightly lower than the MODIS estimate. The MODIS‐based maximum estimate agreed well with the Sentinel‐2 estimate (slope = 0.89, *R* ^2^ = 0.77), whereas the MODIS‐based best estimate tended to underestimate fire‐related forest loss (slope = 0.64, *R* ^2^ = 0.74; Figure S15). Lower estimates for both MODIS and VIIRS can be attributed to omission errors because of missed small fire detections (Table S1). On the other hand, for the maximum estimates the coarser sensor resolution caused considerable commission errors, which largely balanced out against the omission errors for regional to continental‐scale estimates. In primary humid tropical forests, the MODIS‐based estimate of fire‐related forest loss was 22 ± 14% compared to 17 ± 2% for Sentinel‐2. For the rest of Africa, including several frequently burning regions, this was 30 ± 15% for MODIS compared to 42 ± 3% for Sentinel‐2. The MODIS‐based maximum estimate (45%) was closest to Sentinel‐2 outside of primary humid tropical forests, but substantially overestimated the fraction of fire‐related forest loss (36% for MODIS compared to 17% for Sentinel‐2) inside these forests.

## 4 DISCUSSION

### 4.1 Fraction of fire‐related forest loss

Of the global forest loss during 2003–2018, 38 ± 9% was fire‐related and this fraction explained 89 ± 5% of the interannual variability in global forest loss rates. This suggests that drivers involving fire, including wildfire and human‐induced fires for land‐use conversion, regulate variability on annual time‐scales. Weather conditions, including droughts, can synchronize wildfire activity in the boreal forest regions (de Groot et al., 2013) but also the timing of agricultural burning and forest clearing fires, leading to simultaneous peaks in burned area and forest loss (Chen et al., 2017; van der Werf et al., 2008). In contrast, non‐fire drivers tend to respond more slowly, for example as a consequence of longer‐time‐scale socio‐economic factors that impact forestry.

Our estimate of fire‐related forest loss is considerably higher than previous comparable estimates (Liu et al., 2019; Pugh et al., 2019), much of which can be attributed to differences in the approach taken, and particularly the inclusion of active fires in our study. Based on the same forest loss and burned area datasets, Liu et al. (2019) found that 15% of global forest loss in 2003–2014 was fire‐related. In comparison, for the same time period, our estimate based on only burned area detections was 22% (compare figure 1a,b in Liu et al. (2019) to Figures S7f and S9a in our study). The different estimates can be predominantly explained by differences in approach: Liu et al. omitted weighting by forest loss area when spatiotemporally averaging fractions of fire‐related forest loss (Z. Liu, private communication), whereas we included forest loss area as a weighting factor in each 500 m pixel. Furthermore, Liu et al. masked out pixels with less than 20% fraction tree cover after aggregation of their results to a 2° × 2° grid, whereas we applied the masking at the native 30 m resolution of the forest loss data. The impact of the remaining methodological differences between studies was captured by our uncertainty range of ±2% for the part of the fire‐related forest loss estimate that was based on burned area detections, and had a minor effect in comparison (see Section 2 and Figure S3). Besides these differences, we show that the inclusion of active fire detections is essential in most subtropical and tropical regions, and interior tropical forests in particular (Figures S7f, S9, S10c), raising the fire‐related fraction of global forest loss by two‐thirds (to 38 ± 9%) compared to an estimate purely based on burned area.

Our fire‐related forest loss estimate of 38 ± 9% is in line with the results from Curtis et al. (2018), who found that in total 68% of global forest loss is caused by drivers that may involve fire (commodity‐driven deforestation 25%, shifting agriculture 21%, wildfire 22%). Depending on the extent to which deforestation and shifting agriculture involve fire, their results give a range of fire‐related forest loss between 22% (only wildfire fire‐related) and 68% (assuming also all deforestation and shifting agriculture are completely fire‐related). Further determination of the fraction of fire‐use for specific forest loss drivers is required to improve this comparison.

Although the fraction of fire‐related forest loss in Africa was considerable, estimates in our study may be lower than expected considering the dominant role of slash‐and‐burn shifting agriculture on the continent. Curtis et al. (2018) estimated that 92% of forest loss had shifting agriculture as a driver, and two other studies that focussed on the DRC and CAR found that 80%–90% of forest loss was driven by shifting agriculture there (Tyukavina et al., 2018; Zarin et al., 2016). In comparison, we estimated a fire‐related forest loss fraction of 37 ± 22% over 2003–2018 for the DRC and CAR. The higher estimate based on Sentinel‐2 burned area indicates that this can partly be attributed to missed fire detections. Fire detections can be hindered because of persistent cloud cover, dense canopy cover (in the case of understory fires; Morton et al., 2013), small fires or short‐lived fires (e.g. small‐holder agriculture; Giglio et al., 2006; Randerson et al., 2012; Schroeder et al., 2008). Furthermore, the difference could be an indication that shifting agriculture not always involves the use of fire, although further research is required to clarify this. This highlights that our estimate of fire‐related forest loss is likely conservative in this region and associated with considerable uncertainty, due to difficulties regarding fire detection and the spatial and temporal resolution mismatch between fire and forest loss data products. The strongly fragmented and small‐scale forest loss in Africa (Figure S4a) made a related burned area detection unlikely and the spatial overlap with active fire detections uncertain (expressed by low values of $P_{\min}$, see Equation 5), resulting in the largest relative uncertainty in our fire‐related forest loss estimate of any continent.

Fire‐related forest loss in Southeast Asia, and Indonesia in particular, is likely to be underestimated for similar reasons as for Africa. Besides the common limitations in detecting fires in tropical forest regions, thick smoke and haze from peat fires have been shown to obstruct active fire detections in this region (Atwood et al., 2016). However, when compared to the other main deforestation region, that is, the Amazon, non‐fire‐related forest loss is more prominent in Indonesia. This is at least partly due to the large‐scale development of tree plantations, both for logging and palm oil (Abood et al., 2015; Noojipady et al., 2017), also indicated by substantial forest gain (Figure S4c).

Differences in fire‐related forest loss across Siberia highlight a boundary between a non‐stand‐replacing fire regime in Southern Siberia, where human influence is significant, and a stand‐replacing wildfire regime further north (Krylov et al., 2014). Similarly, continental differences in the role of fire in boreal forest loss (larger role in North America, lesser role in Asia) could be explained by differences in fire type, with more high severity canopy fires in boreal North America and more low severity surface fires in boreal Asia (Rogers et al., 2015). These continental differences in fire severity could also explain why there is more overlap of forest loss detections with fires from the preceding year in boreal Asia as compared to boreal North America (Figure S2; months −12 to 0). Lower fire severity could lead to longer delays in post‐fire tree mortality and delayed forest loss detection. Understanding these regional and continental differences in fire‐related forest loss is critical for interpreting future changes in boreal forests.

### 4.2 Trends in the fraction of fire‐related forest loss

On a global level, the fraction of fire‐related forest loss remained stable over the study period. Regionally, however, we found marked declines in this fraction for older deforestation frontiers in South America (primarily Amazon forests) and Southeast Asia (primarily Sumatra and Kalimantan). These declining trends were particularly strong for primary humid tropical forests (Figure 4). In the Amazon, deforestation activity peaked around 2004 and declined afterwards (Turubanova et al., 2018). We observed a parallel decline in the fraction of fire‐related forest loss (Figure 2). This could signify a change in the dominant forest loss driver from commodity‐driven deforestation to drivers that are less related to fire (e.g. forestry, mining; Asner et al., 2013), or fires in smaller clearings (Rosa et al., 2012) and in the forest understory (Morton et al., 2013) that are more difficult to detect. The 2015−2017 period was marked by a resurgence of the role of fire in forest loss that may be partially linked to the exceptionally strong 2015–2016 El‐Niño event which led to increases in drought‐induced forest fires (Chen et al., 2017). These fires have been found to constitute more than half of the forest loss emissions from the Brazilian Amazon in 2015 (Aragão et al., 2018; Silva Junior et al., 2019). Likewise, the peak in 2010 could be linked to the earlier 2010 drought event. In addition, a gradual increase in recent deforestation might also have contributed to the resurgence from 2015 onwards (Barlow et al., 2020).

A similar course was found for Indonesia. After a decline from historic deforestation rates, a peak in the fraction of fire‐related forest loss was visible for 2015–2016 that might be related to peat fires during the exceptional 2015–2016 El Niño (Figure 2b; Atwood et al., 2016; Silvius et al., 2018). Additionally, fire‐related forest loss rapidly declined after 2016 in this region. This could be related to legislation changes in Indonesia regarding plantation expansion, with the goal to reduce forest loss, suppress fires and restore peatlands, accompanied by relatively wet weather conditions associated with La Niña (Carlson et al., 2018; Gaveau et al., 2019). A longer time record is required however, including drought years, to test whether this decline is permanent.

Spatial patterns for trends in the fraction of fire‐related forest loss across sub‐Saharan Africa largely coincided with trends in burned area and active fire detections (Figure 2; Figure S8). Global burned area in low tree cover systems, that is, low fuel consumption fires, has been shown to be decreasing in the last two decades (Andela et al., 2017). We found related decreasing trends in the fraction of fire‐related forest loss in relatively low tree cover areas. However, we also observed increases in forest loss and active fire detections in high tree cover areas such as the Congo basin, where the efficacy of burned area detection is low. These related patterns between forest loss and fires could reflect an increase in human influence (Andela & van der Werf, 2014), expressed as a reduction of large fires in natural lands (often detected as burned area) in combination with an increase in deforestation fires and/or small‐scale shifting agriculture in the African tropical forests (both commonly only detected as active fires; Tyukavina et al., 2018). The increase in fires in high tree cover areas, constituting a disproportionally large part of fire emissions (van der Werf et al., 2017), could gain importance in determining global emissions in the future.

Climate change is expected to especially affect fire activity in the temperate and boreal regions, by lengthening the fire season, shortening fire return times, creating drier fuel conditions and increasing lightning activity (Jolly et al., 2015; Veraverbeke et al., 2017; Williams et al., 2019), which could, in turn, drive additional forest loss. However, we did not find significant trends in the fraction of fire‐related forest loss for the temperate and boreal regions over our study period (Table 1). A longer record of forest loss and fire detections is required to adequately capture the multi‐decadal fire return intervals characteristic of these fire regimes. However, we did find that 89 ± 5% of the interannual variability in global forest loss rates was fire‐related, indicating that fire‐prone regions might experience a further acceleration of forest loss with warming climate while other forested regions where fires are less frequent might be more resilient. This is also relevant for humid tropical forests, where prolonged drought periods under climate change increase the risk of managed fires escaping into adjacent forest, driving tropical forest degradation, especially during El‐Niño events (Brando et al., 2019; Cai et al., 2014; Jiménez‐Muñoz et al., 2016; Phillips et al., 2009).

### 4.3 Comparison to higher‐resolution data

The similarity between fire‐related forest loss estimates based on MODIS versus VIIRS active fires indicates that the MODIS‐based approach is capable of extending the analysis to the pre‐VIIRS period (2003–2011) with only a minimal reduction in accuracy as compared to the higher‐resolution VIIRS‐based approach (Figure 2b). In regionally averaged estimates, commission errors due to the larger fire pixel size for the MODIS sensor largely balanced omission errors due to its lower detection efficiency (Table S1). Commission errors were limited because of a rapid decrease in $P_{\min}$ in Equation 3 in cases of overlap between large, off‐nadir fire pixels and small forest loss areas (Figure S12a). For the maximum estimate, commission errors were generally less than expected, given the 11‐fold larger fire pixel footprint of MODIS compared to VIIRS. This was the case because forest loss areas in 500 m pixels were often sufficiently spatially separated or in larger continuous fire‐affected regions to avoid major commission error for the larger MODIS active fire pixel footprint (see also Figure S1).

Differences between the MODIS and VIIRS sensor were also analysed temporally as part of the lag analysis. Within a single year, MODIS active fire pixels overlapped with about 25% more forest loss than VIIRS on average (Figure S16). However, this did not translate into similarly large commission errors in annual maximum fire‐related forest loss estimates, because over the span of a year, the MODIS active fire pixels had overlapped each other more than what would have been the case for the smaller VIIRS pixels. VIIRS‐based fire‐related forest loss in years preceding and succeeding the forest loss detection was smaller compared to MODIS‐based estimates, due to lower commission error in lag years.

The substantial increase in spatial resolution and amount of burned area detected using the Sentinel‐2 MSI sensor (80% more burned area than MODIS for 2016 sub‐Saharan Africa; Ramo et al., 2021) led to a higher fire‐related forest loss fraction due to improved small fire detection, especially in heavily cultivated, frequently burning regions with relatively low tree cover (i.e. woodlands). However, this did not result in a larger attribution in most densely forested areas. Compared to the Sentinel‐2 ‐based estimate, the MODIS‐based estimate actually tended to be higher in this region due to commission errors caused by the coarser resolution fire pixels (Figure S15). Besides, it suggests that the 20 m Sentinel‐2 burned area product fails to detect part of the fires in the forest interior that are detected as active fires. Overestimation by our MODIS‐based approach can be expected for other regions where shifting agriculture is an important forest loss driver, such as parts of South America and Southeast Asia. However, the comparison to Sentinel‐2 burned area shows that the overestimation by our MODIS‐based approach in such regions is largely compensated for by the underestimation due to missed fire detections.

Overall, the spatial and temporal resolution differences between the forest loss and fire datasets were the main source of uncertainty in our estimate. Comparison to Sentinel‐2 burned area showed that omission errors due to missed fire detections can be substantial for the coarser MODIS and VIIRS fire products, especially in the tropics. Therefore, the estimated fraction of fire‐related forest loss in these regions is likely to be conservative. At the same time, the use of the coarser resolution MODIS and VIIRS active fire pixels can lead to substantial commission errors. However, using fire data with very different spatial resolutions, we show that omission and commission errors largely balanced out and resulted in comparable fire‐related forest loss estimates on regional to continental scales.

### 4.4 Further application of our methodology

Our study provides a novel approach for the quantitative analysis of fire‐related forest loss that allows for a consistent simultaneous analysis of dynamics in fire and forest loss. Furthermore, this approach improves on distinguishing between different fire types in forests (low or high severity) that can be applied to fire emissions modelling at 500 m native MODIS resolution (van Wees & van der Werf, 2019). By combining datasets of annual forest loss and monthly fires, emissions from forest loss can be distributed at finer temporal scales, allowing improved seasonality in models that simulate the atmospheric dynamics of CO_2_ and other trace gases. These advances could help in clarifying the outcome of the balance between forest loss and fire into the future, and in improving estimates of global land‐use change emissions. Currently, version 1.6 of the GFC dataset does not allow for the analysis of trends in absolute forest loss and fire‐related forest loss directly, or analysis of forest loss drivers in detail, due to data inconsistencies (see Data S1). However, recent developments in forest loss detection methodology demonstrate the possibility of a consistent forest loss time series that is suitable for trend analysis, and that could potentially be applied to global scale (Potapov et al., 2019; personal communication). This would further improve the analysis of dynamics in forest loss, fire and their interface.

## AUTHORS’ CONTRIBUTIONS

D. van Wees and G.R. van der Werf designed the study concept, D. van Wees designed the methodology and performed all analyses, D. van Wees wrote the manuscript with contributions from G.R. van der Werf, J.T. Randerson, N. Andela, Y. Chen and D.C. Morton. All the authors contributed to the interpretation of the results and the refinement of the manuscript.

## Supporting information

Supplementary Material

## Data Availability

The data that support the findings of this study are available from the corresponding author upon reasonable request.
