# Supplementary material for: The role of fire in global forest loss dynamics
Source: Glob Chang Biol. 2021 Mar 27;27(11):2377–91. doi: 10.1111/gcb.15591 (PMC8251961; doi:10.1111/gcb.15591)

Supplementary Materials for

The role of fire in global forest loss dynamics

Dave van Wees, Guido R. van der Werf, James T. Randerson, Niels Andela, Yang Chen, Douglas C. Morton

Correspondence to:
Dave van Wees (d.van.wees@vu.nl)
Guido van der Werf (g.r.vander.werf@vu.nl)

Published in Global Change Biology
DOI: https://doi.org/10.1111/gcb.15591

This PDF file includes:

- Sensitivity of fire-related forest loss estimate to active fire scan geometry
- Time series inconsistencies in the GFC version 1.6 dataset
- Table S1
- Figures S1 to S16

Sensitivity of fire-related forest loss estimate to active fire scan geometry

The pixel size of detections in the used active fire products is dependent on the scan angle and the associated along-scan and along-track pixel dimensions. This has to be considered with regard to our fire-related forest loss estimate, and in particular the sensitivity of P_{min} (Eq. 3). The terms A_f and C_f in Eq. 2 implicitly include the reduced confidence of active fire detections towards the scan edge. Figure S6 shows that for larger scan angles the reduction in $P_{single\ fire}$ is predominantly driven by the reduction in C_f. This indicates that the increase in A_f is balanced by the increase in forest loss area in the larger active fire pixel, which makes our approach relatively insensitive to scan angle effects and the spatial resolution of the used active fire products.

The impact of latitude-dependent detection differences should also be considered. Relatively speaking, more active fires are detected towards higher latitudes because consecutive satellite orbits converge towards the poles. This could potentially result in a latitude bias in the calculated probability of fire-related forest loss. Figure S7 shows how these latitude-dependent factors play out for the value of P_{min}. The detection confidence, C_f, weighted by forest loss area is relatively stable over latitudes, ranging from 0.6 to 0.8 on average for all active fire detections. This is also the case for average active fire pixel area weighted by forest loss area, with an average value of about 3 km^2 over all latitudes. This indicates that both $P_{single\ fire}$ and P_{min} (Eq. 3) are relatively insensitive to meridional differences in the active fire scan geometry. Instead, P_{min} is mostly determined by the amount of forest loss in an active fire pixel and the number of fire detections overlapping forest loss, as intended.

Time series inconsistencies in the GFC version 1.6 dataset

Trends in 2003-2018 forest loss from the Global Forest Change (GFC) version 1.6 dataset (Hansen et al., 2013) are affected by inconsistencies in the dataset time series, and therefore we focus our trend analysis on the relative change in fire-related forest loss instead of absolute forest loss values (https://earthenginepartners.appspot.com/science-2013-global-forest/download_v1.6.html). First, the forest loss detection methodology has changed over time, which results in different detection rates for the periods of 2001-2012, 2013-2014, and 2015-present. Methodological changes include the introduction of Landsat-8 OLI images from 2013 onwards and improvements in change detection for the three periods for boreal forest fires, smallholder agricultural clearing in tropical forests, selective logging, and clearing of short cycle plantations. These changes generally result in a relative overrepresentation of detections for the later time period and thus an artificial increase. Second, each 30 m grid pixel is limited to only one forest loss detection over the full time series, resulting in the omission of repeated events such as slash and regrowth cycles typical for shifting agriculture, and an associated temporal bias. We limited our analysis to the relative contribution of fire to forest loss, instead of absolute forest loss area, in order to be largely unaffected by these methodological changes.

Table S1: Percentage of omission (OE) and commission (CE) error for minimum-, best-, and maximum-estimate fire-related forest loss as compared to higher resolution fire products. Comparisons of MODIS versus VIIRS are shown for the regions in Table 1 of the main text. Comparisons of MODIS versus Sentinel-2 MSI, and VIIRS versus Sentinel-2 MSI are shown for Northern-hemisphere Africa (NHAF), Southern-hemisphere Africa (SHAF), and the continent as a whole.

	**Minimum estimate**		**Best estimate**		**Maximum estimate**	
	OE (%)	CE (%)	OE (%)	CE (%)	OE (%)	CE (%)
**MODIS – VIIRS**						
Global	9	9	13	17	15	22
Middle & South America	17	15	21	26	24	32
Africa	12	8	21	25	25	31
Southeast Asia	22	18	26	32	27	38
Boreal	4	7	4	7	4	8
Temperate	6	5	10	18	13	28
Amazon	22	15	24	25	25	31
Indonesia	24	22	28	34	30	40
**MODIS – Sentinel**						
Africa	74	13	51	28	40	55
NHAF	76	12	55	27	43	52
SHAF	73	14	49	29	38	58
**VIIRS – Sentinel**						
Africa	73	12	53	27	43	50
NHAF	74	10	56	27	46	49
SHAF	71	13	50	27	41	51

Figure S1: Regional examples of 500 m MODIS grid fraction forest loss (left panels) and overlap with fire detections (right panels), for MODIS burned area and active fire detections in the year of forest loss (current) and the preceding year (previous). In case multiple fire detection types overlap forest loss in one 500 m pixel, the type of data overlap furthest right on the colorbar is displayed, i.e. burned area is favored for display over active fire and detections from the current year are favored for display over detections from the preceding year. **(a)** Central Africa transition from Congo rainforest to savanna in 2017 (central coordinate: 3°12'38"S 26°55'10"E), **(b)** Western Africa transition in 2014 (central coordinate: 7°29'53"N 8°49'24"W), **(c)** Deforestation on Sumatra in 2005 (central coordinate: 0°12'23"N 102°11'25"E), and **(d)** Canadian wildfires around Great Slave Lake in 2014 (central coordinate: 61°39'53"N 117°37'50"W).

(a)

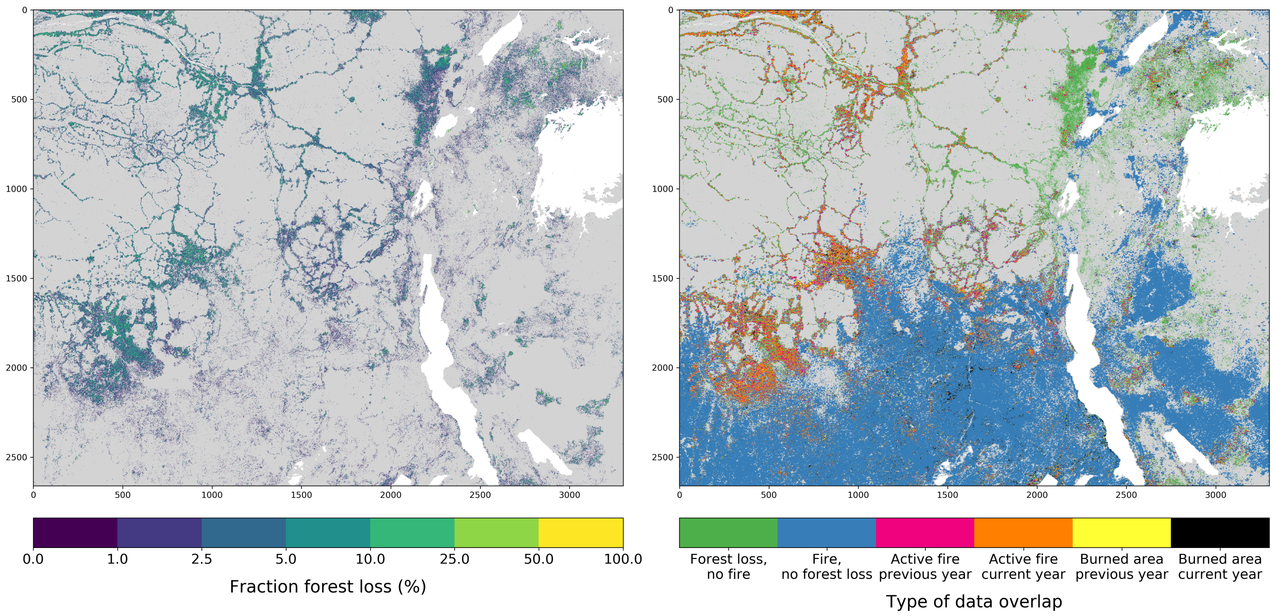

(b)

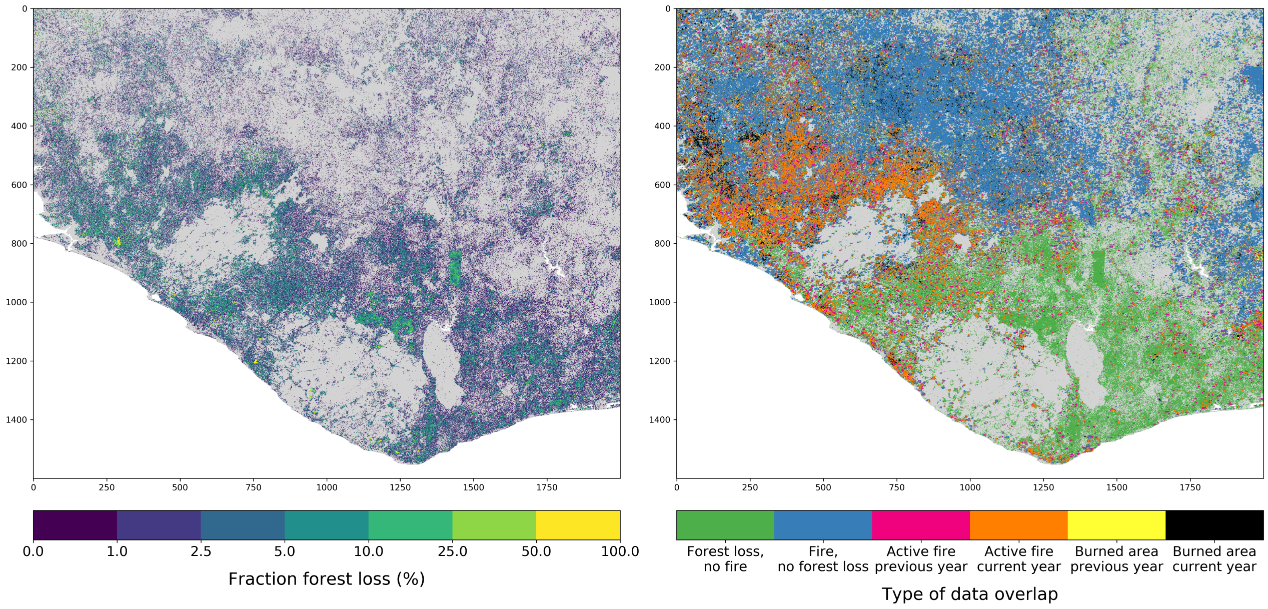

(c)

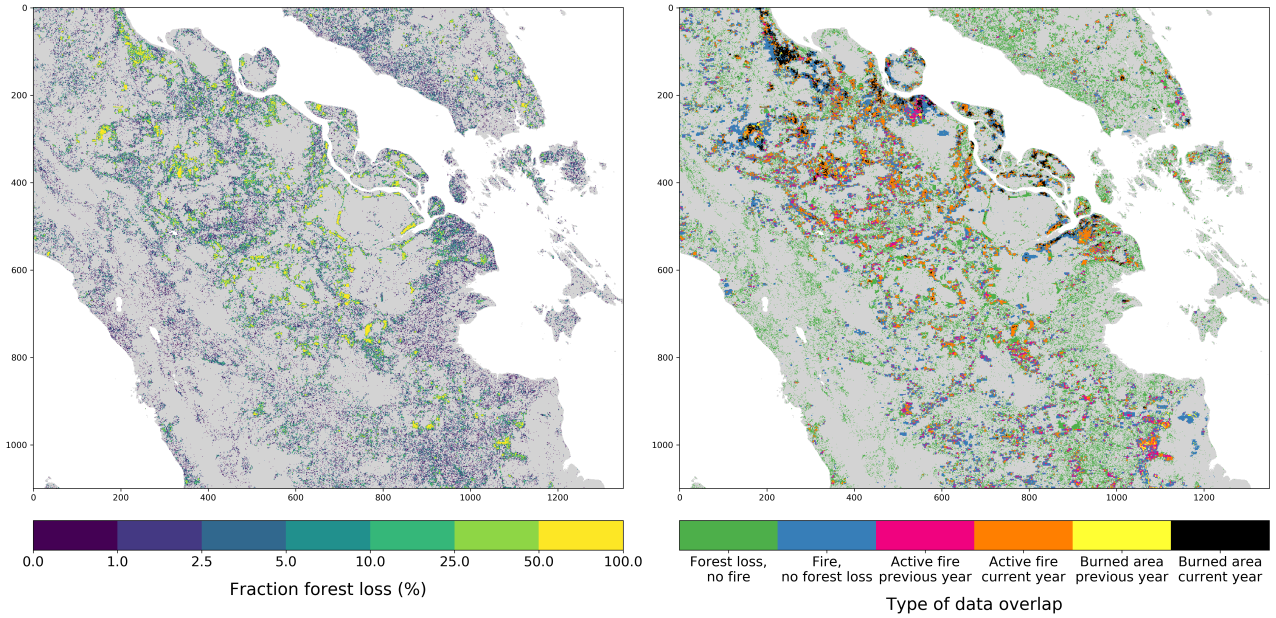

0
200
400
600
800
1000
1200
0.0
1.0
2.5
5.0
10.0
25.0
50.0
100.0
Fraction forest loss (%)
Forest loss, no fire
Fire, no forest loss
Active fire previous year
Active fire current year
Burned area previous year
Burned area current year
Type of data overlap

(d)

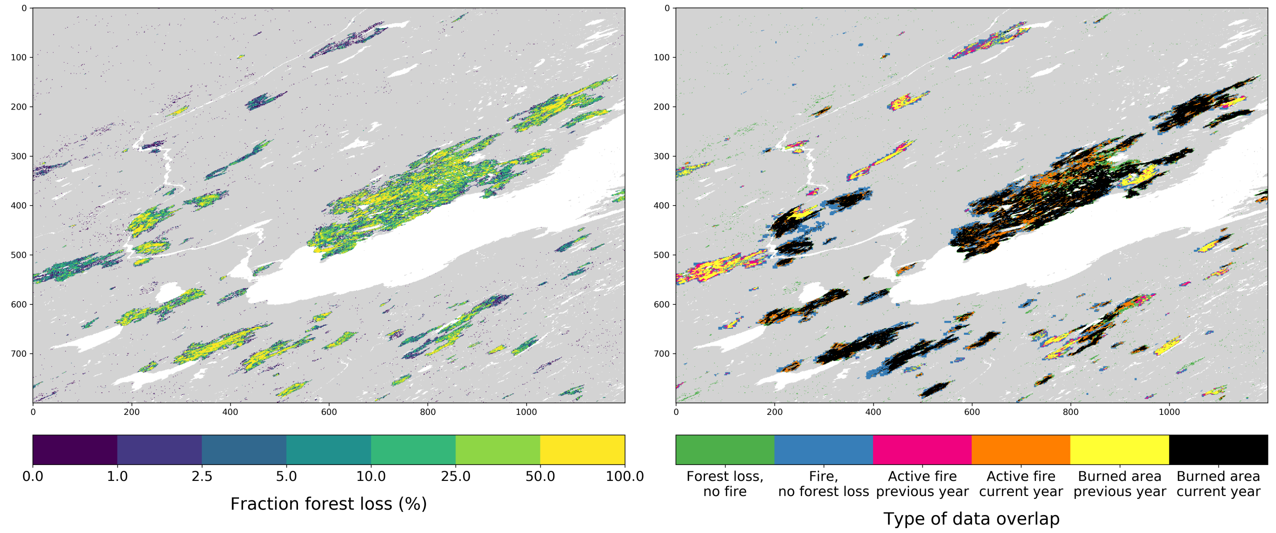

0
100
200
300
400
500
600
700
800
1000
0.0
1.0
2.5
5.0
10.0
25.0
50.0
100.0
Fraction forest loss (%)
Forest loss, no fire
Fire, no forest loss
Active fire previous year
Active fire current year
Burned area previous year
Burned area current year
Type of data overlap

Figure S2: Lag analysis of monthly fire detections overlapping forest loss on 500 m pixel level, for fire detections from 24 months before until 24 months after the forest loss event. Histograms show monthly overlap of 2004-2016 forest loss with MODIS burned area and active fires, for the global average and the 14 GFED regions (see Figure 3 in van der Werf et al., 2017). Displayed fire-related forest loss based on active fire detections constitutes only active fire pixels outside of burned area, i.e. total fire-related forest loss minus the part by burned area. Monthly distributions per year were calculated by determining the spatial overlap of forest loss in year $t = 0$ (months 0 to 11) with monthly fires from years $t = -2$ to $t = +2$ (months -24 to 24) before and after the forest loss detection year. The resulting distributions for each forest loss year were then summed and divided by the number of years to create the annual-average distributions as displayed. Percentages displayed above each year (each 12 months) give the amount of fire-related forest loss that is newly added by subsequently incorporating fire detections from lag years, in the order of $t = -1$, $t = -2$, $t = +1$ and $t = +2$, in addition to year $t = 0$. The percentages are displayed as a fraction of the fire-related forest loss from year $t = 0$ and $t = -1$ (months -12 to 11). For example, if the global fire-related forest loss based on fire detections from year $t = 0$ and $t = -1$ is 100%, 32% of that is added by fire detections from year $t = -1$. In turn, 10% is added by year $t = -2$, an additional 8% is added by year $t = +1$, and finally 5% is added by year $t = +2$.

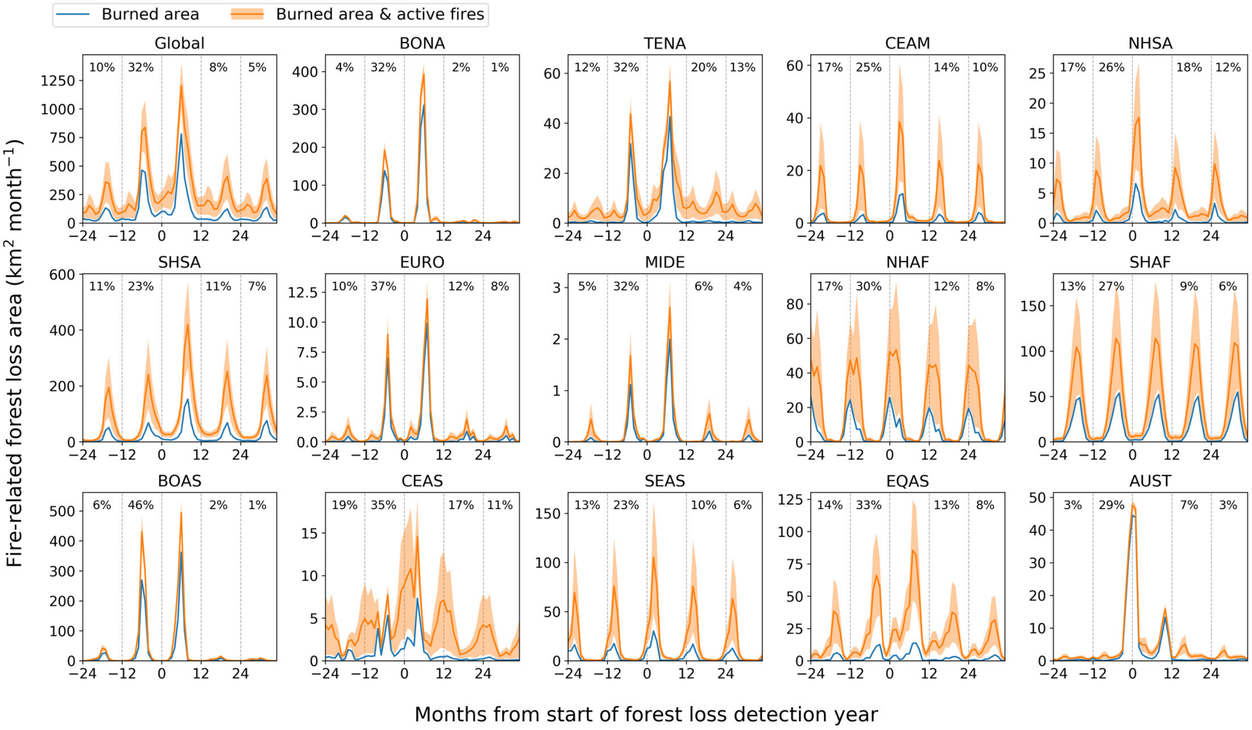

Figure S3: Effects of methodological changes on the fraction of fire-related forest loss estimate based on burned area detections only. **(a)** inclusion of burned area detections from the second preceding year ($t = -2$), additionally to the current and first preceding years ($t = 0$, $t = -1$). **(b)** Exclusion of 500 m pixels containing more than one burned area detection in a single year, instead of involving all detections. **(c)** Exclusion of forest loss in 30 m pixels with less than 20% fraction tree cover.

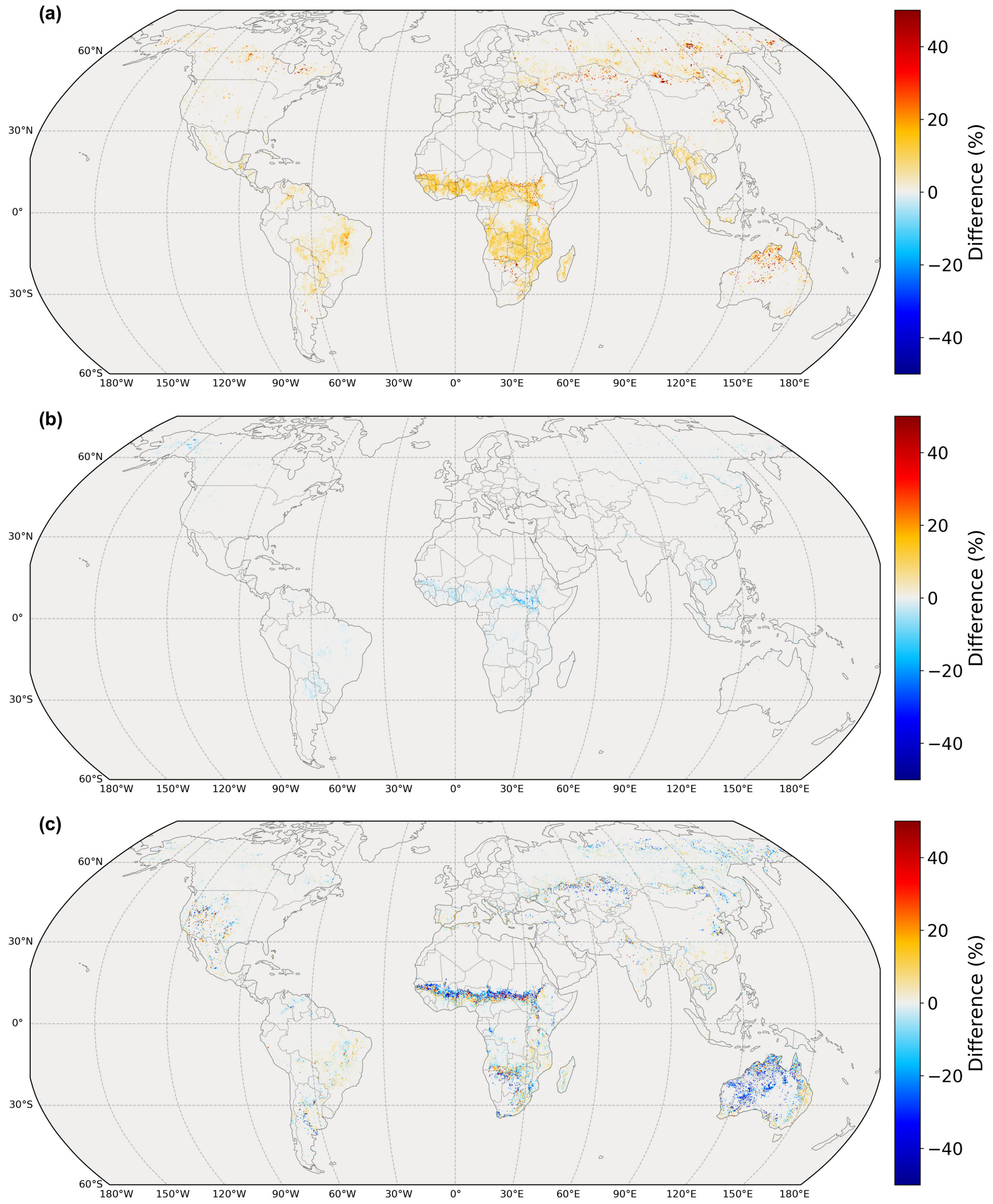

Figure S4: Additional forest loss metrics, **(a)** annual average percentage of forest loss in each 500 m pixel, averaged per 0.25° grid cell (2003-2018). **(b)** Annual average percentage of forest loss in each 500 m pixel for pixels that are overlapped with burned area (BA) detections from the current and first preceding year. **(c)** Percentage of 0.25° grid cell that underwent forest gain from 2000 to 2012.

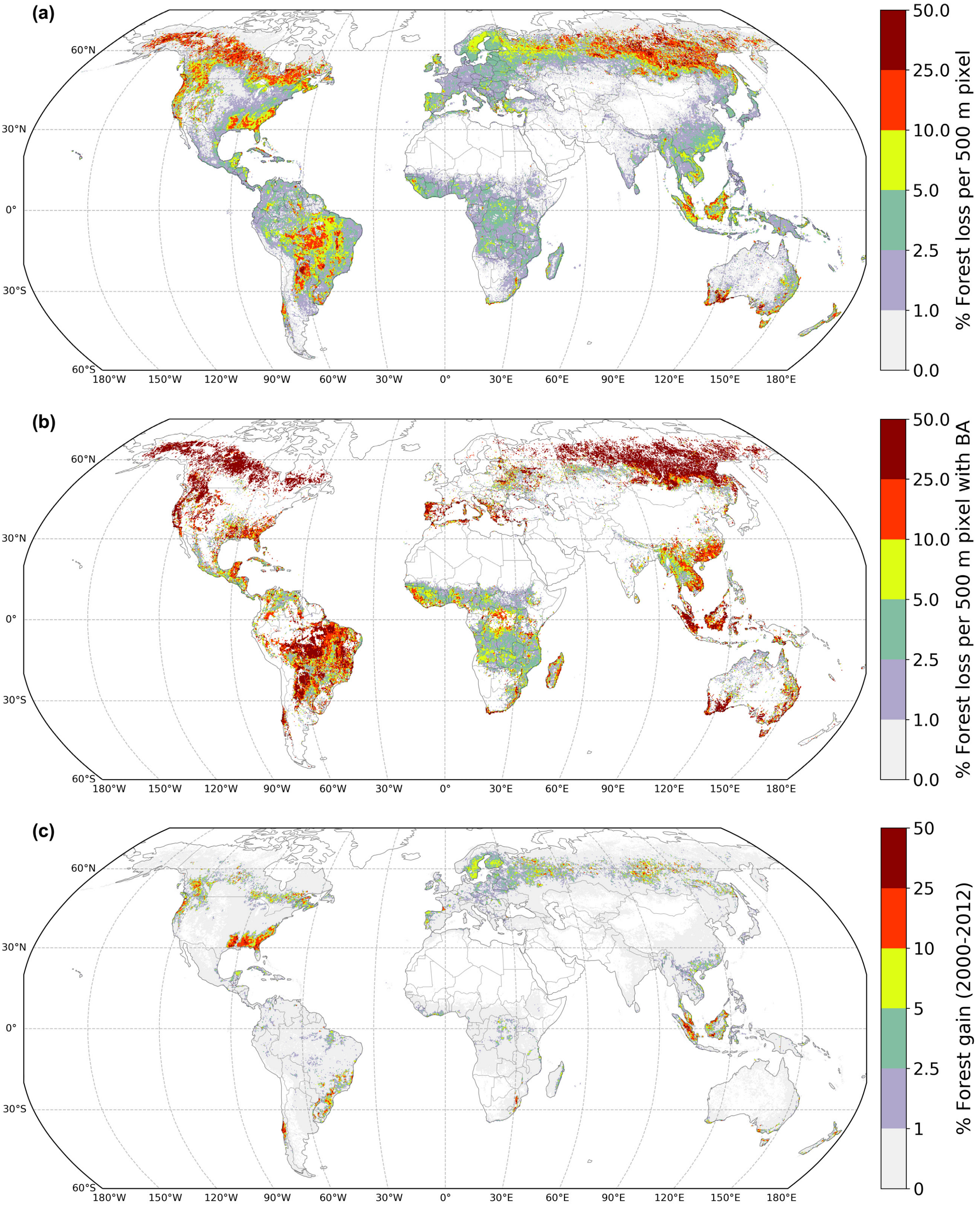

Figure S5: Schematic illustrating the calculation of fire-related forest loss, FL_{fire}, showing an example section of the 500 m grid with the fraction of forest loss per 500 m pixel (as percentages). 500 m pixels that are assigned ‘burned’ based on burned area detections are shown in blue. Active fire pixels projected onto the 500 m grid are demarked by red and yellow borders, and the respective active fire detection confidences, C_f, are given in the bottom-right corner. The factor $\sum_f A_{forest\ loss}$ is the total area of forest loss in the 500 m pixel overlapping a gridded active fire detection (i.e. orbital swath pixel) with area A_f. Example 1, location (2,1): burned area and one active fire detection overlapping forest loss in a 500 m pixel. Here the estimate of fire-related forest loss only depends on burned area; active fires are not considered in this calculation). Example 2, location (3,2): one active fire detection overlapping forest loss and no burned area. Example 3, location (1,2): two active fire detections overlapping forest loss and no burned area. Note that the 500 m^2 pixel size displayed here is a simplification, the actual pixel size is approximately 463 m^2.

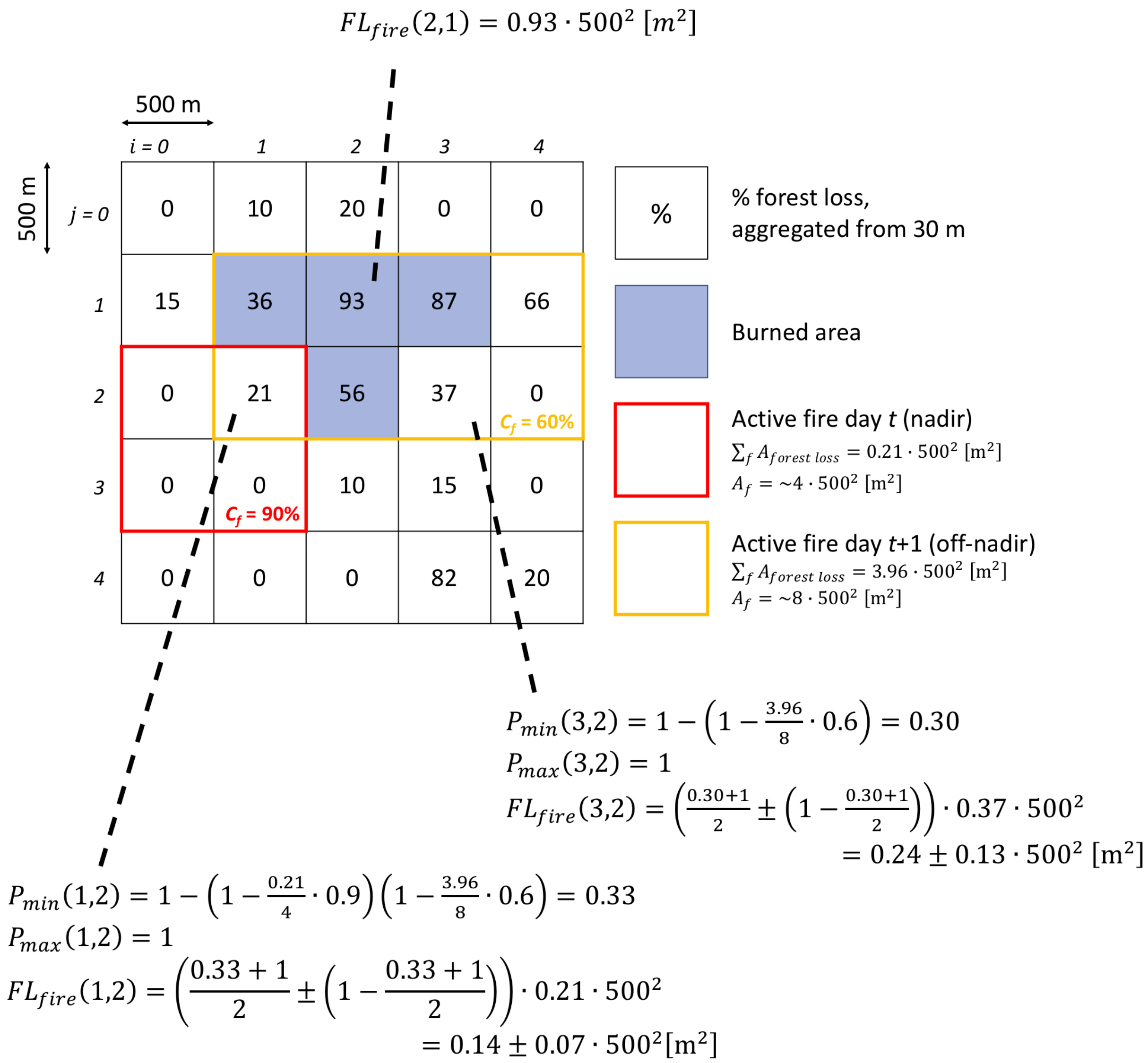

Figure S6: Sensitivity of **(a, b)** the detection confidence, C_f, and **(c, d)** the single-fire probability factor, $P_{single\ fire}$, to the active fire pixel area and scan angle, showing that both variables decrease linearly for larger scan angles. Displayed values are based on 2003-2018 MODIS active fire detections.

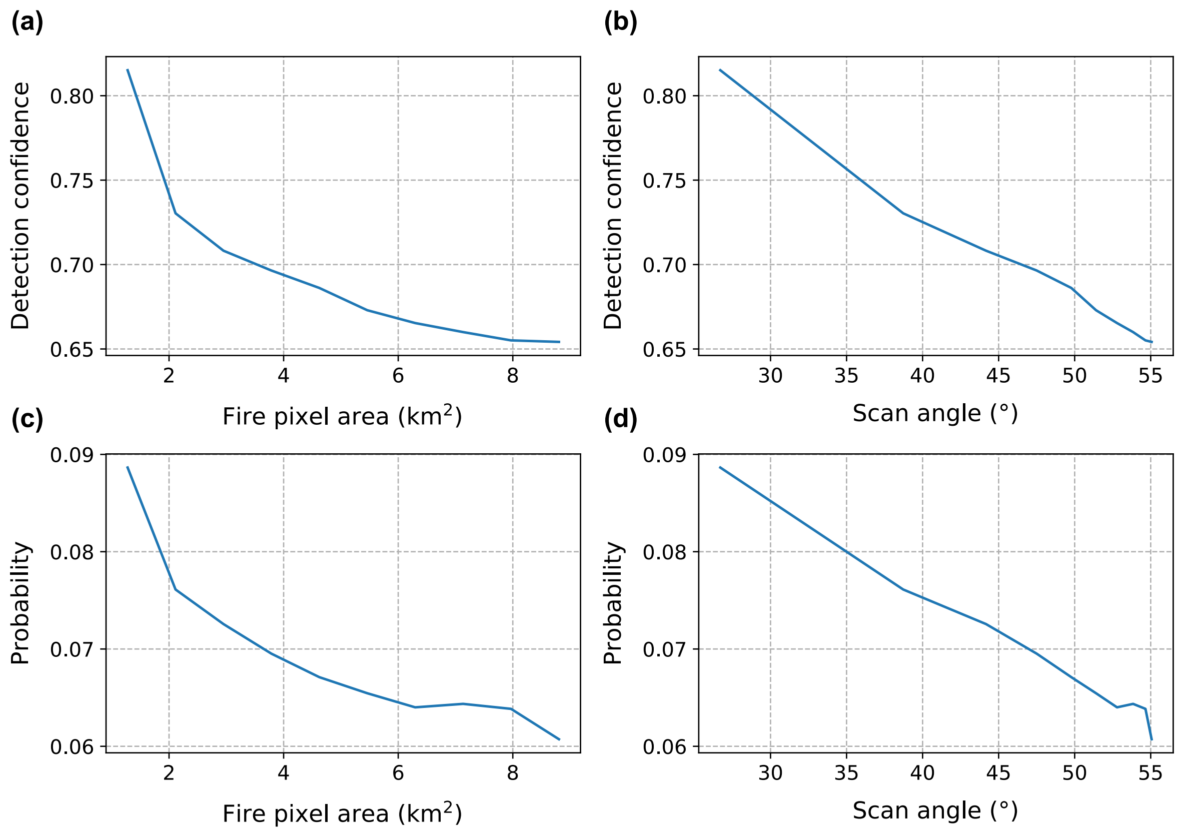

Figure S7: Zonal averages of 2003-2018 **(a)** normalized forest loss, **(b)** normalized number of active fire detections, **(c)** detection confidence per active fire detection, weighted by forest loss area, **(d)** fire pixel area per active fire detection, weighted by forest loss area, **(e)** the value of $P_{single\ fire}$ and P_{min} per active fire detection and weighted by forest loss area, and **(f)** best-estimate fraction of fire-related forest loss and the part due to burned area detections. Transparent filled bands display the range between minimum- and maximum-estimate fire-related forest loss. Values are based on 2003-2018 MODIS burned area and active fire detections. All lines are smoothed using a moving-average filter with a window of n=6 (0.25° · 6 = 1.5°).

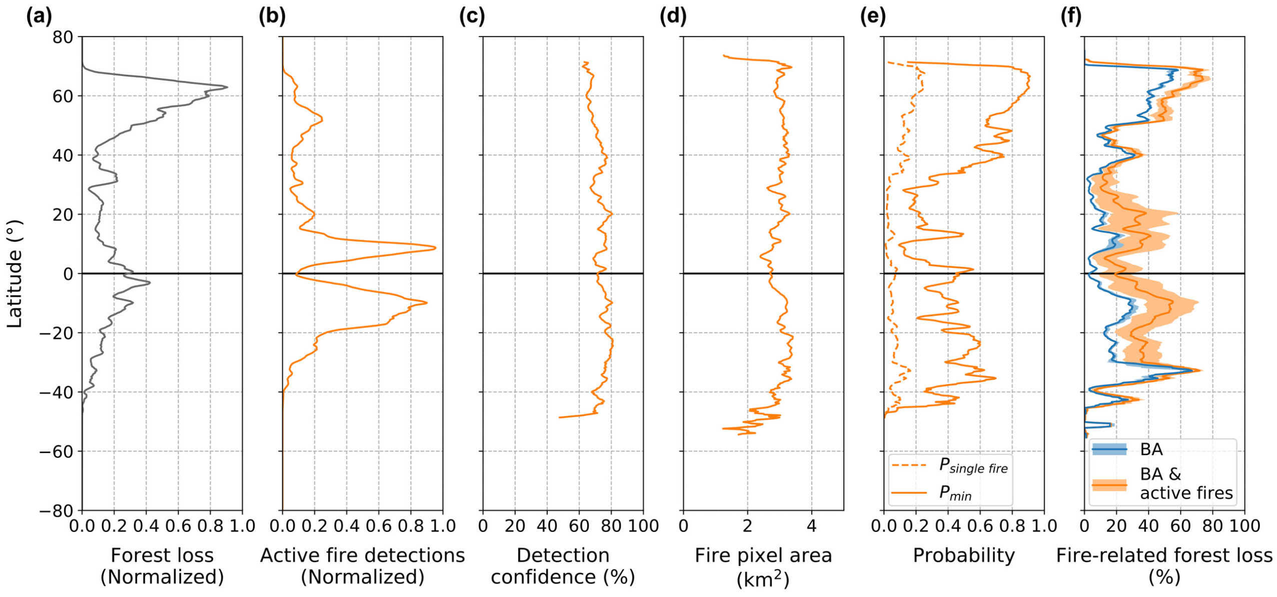

Figure S8: Trends in **(a)** MODIS burned area and **(b)** MODIS active fire detections (for all fire pixels, i.e. in- and outside forests) over the period 2003-2018 on a 0.25° grid.

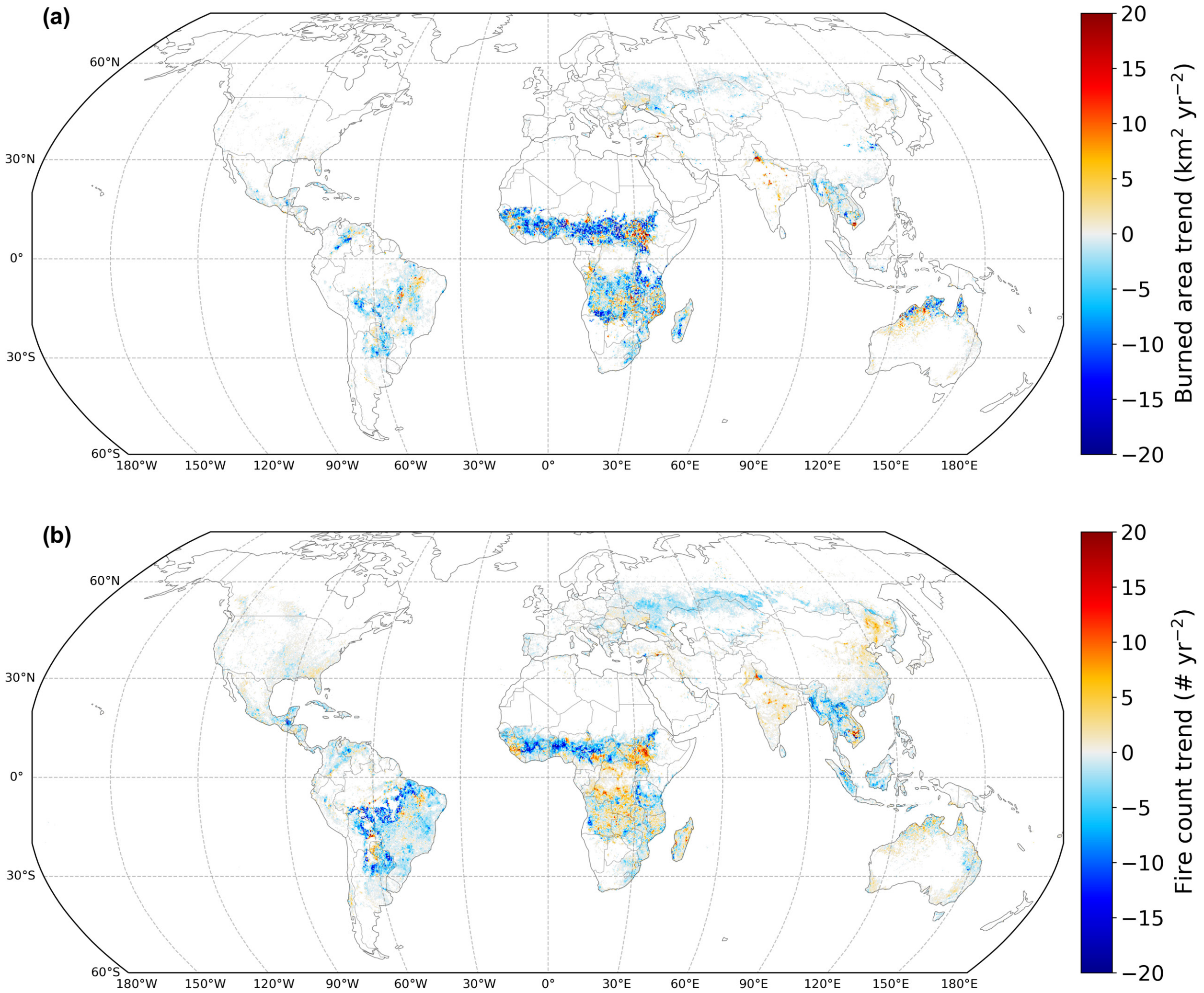

Figure S9: Fraction of fire-related forest loss based on **(a)** MODIS burned area detections only, and additional **(b)** best-estimate and **(c)** maximum-estimate fire-related forest loss due to incorporation of MODIS active fire detections. Aggregated to 0.25° resolution for display. 0.25° grid cells that underwent less than 0.1% forest loss are masked out. The horizontal dimension of the colormap represents the percentage of forest loss from Figure 1a, scaled to the power of 0.5 and clipped at 40% forest loss for improved visualization, analogous to Figure 1b.

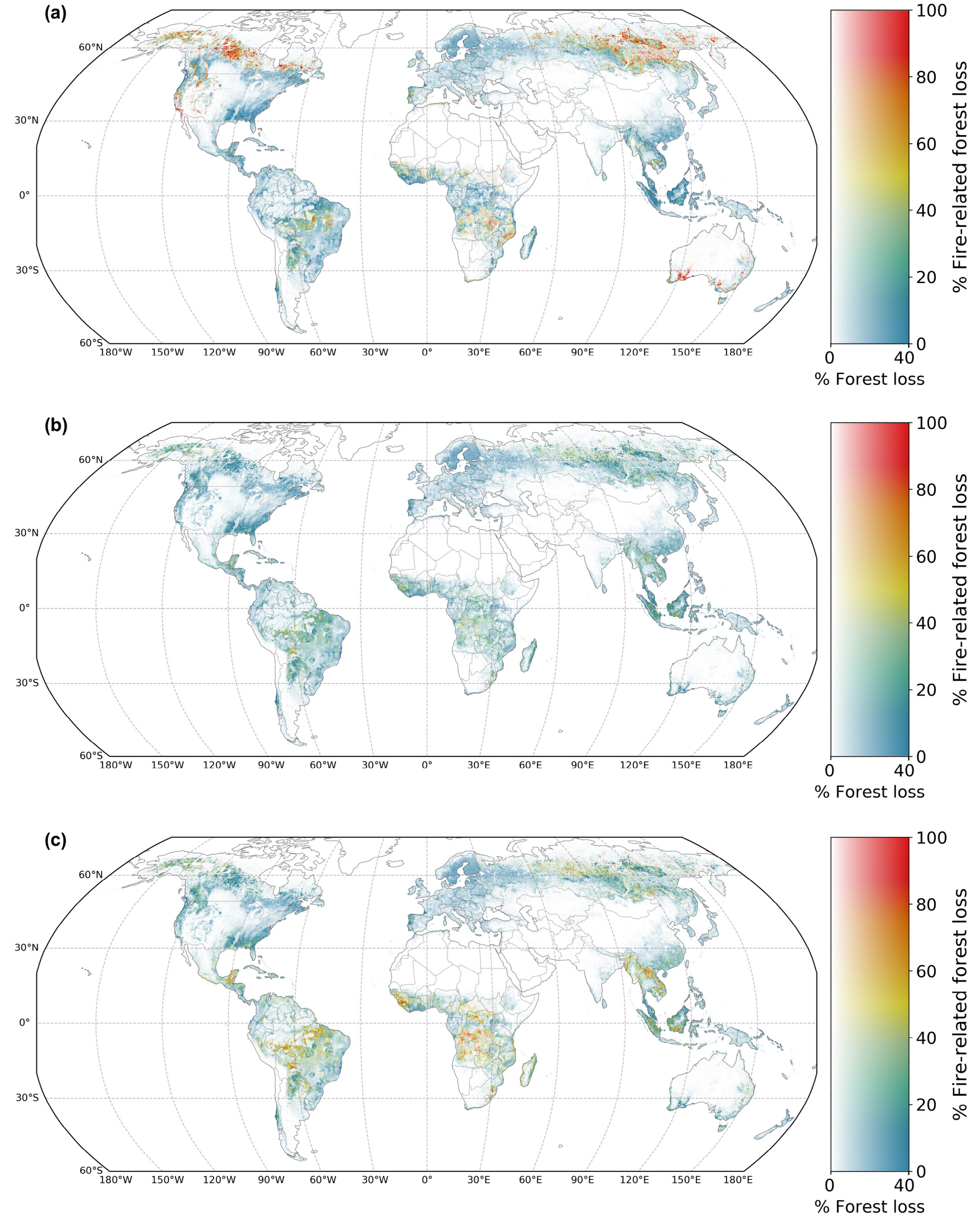

Figure S10: 2003-2018 average fraction of fire pixels overlapping with at least one 30 m forest loss detection in the same year, aggregated to 0.25° resolution for display, for **(a)** burned area and **(b)** active fire detections. **(c)** Fraction of 2003-2018 fire-related forest loss (best estimate) due to active fire detections, showing 0.25° grid cells with more than 0.5% fire-related forest loss. Right-hand panes show the latitudinal average, with the standard deviation given in grey, and the overall global average at the bottom of the pane.

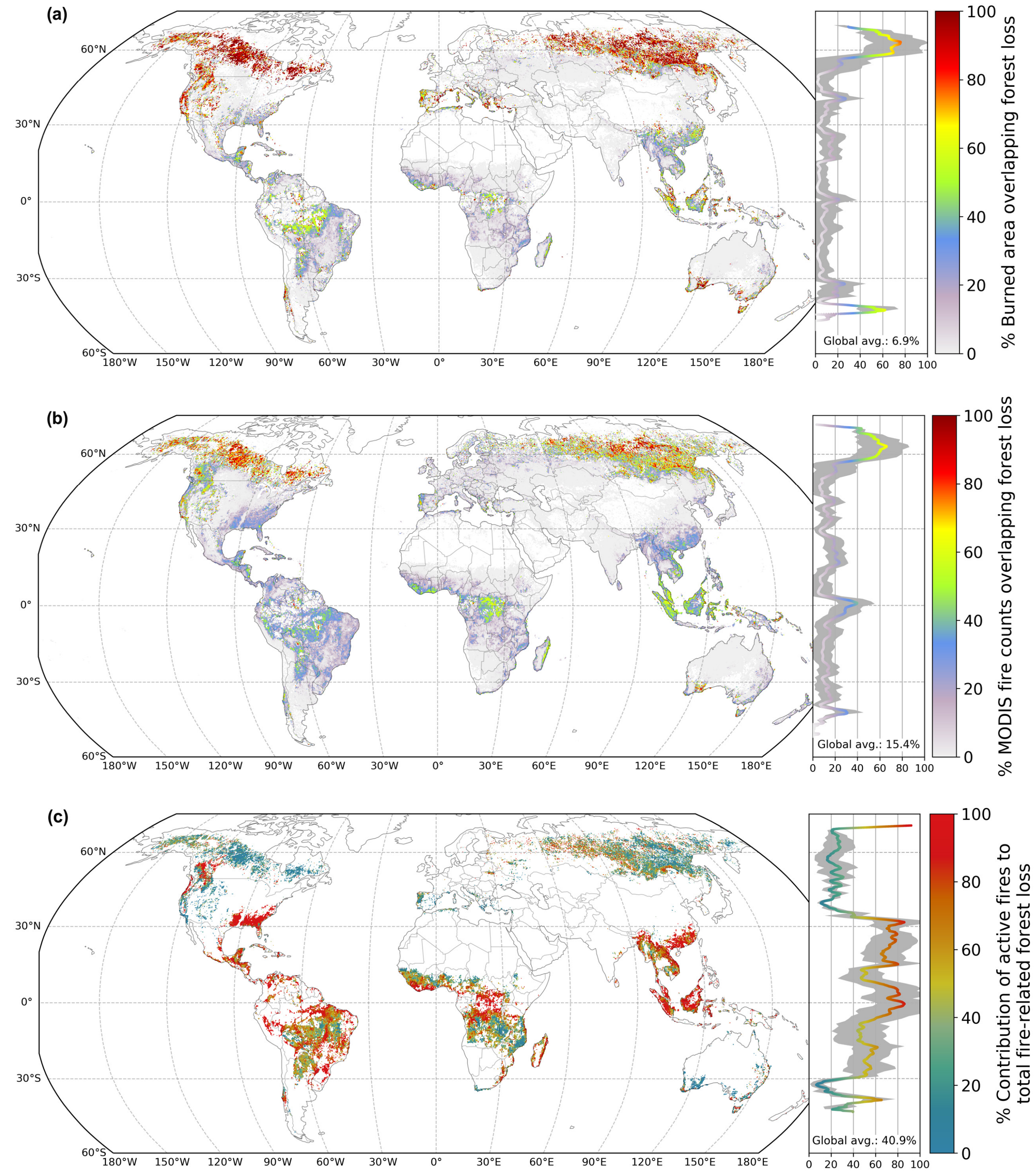

Figure S11: Annual average fire-related forest loss area (2003-2018) based on MODIS for fraction tree cover (FTC) intervals of **(a)** 0-25%, **(b)** 25-50%, **(c)** 50-75%, and **(d)** 75-100%. Bars are separated into the fire-related forest loss contribution by burned area detections (blue) and the additional contribution by active fire detections (orange). The total bar height shows the best estimate fire-related forest loss, with the error whiskers showing the estimate range based on the minimum and maximum fire-related forest loss calculations (see Methods).

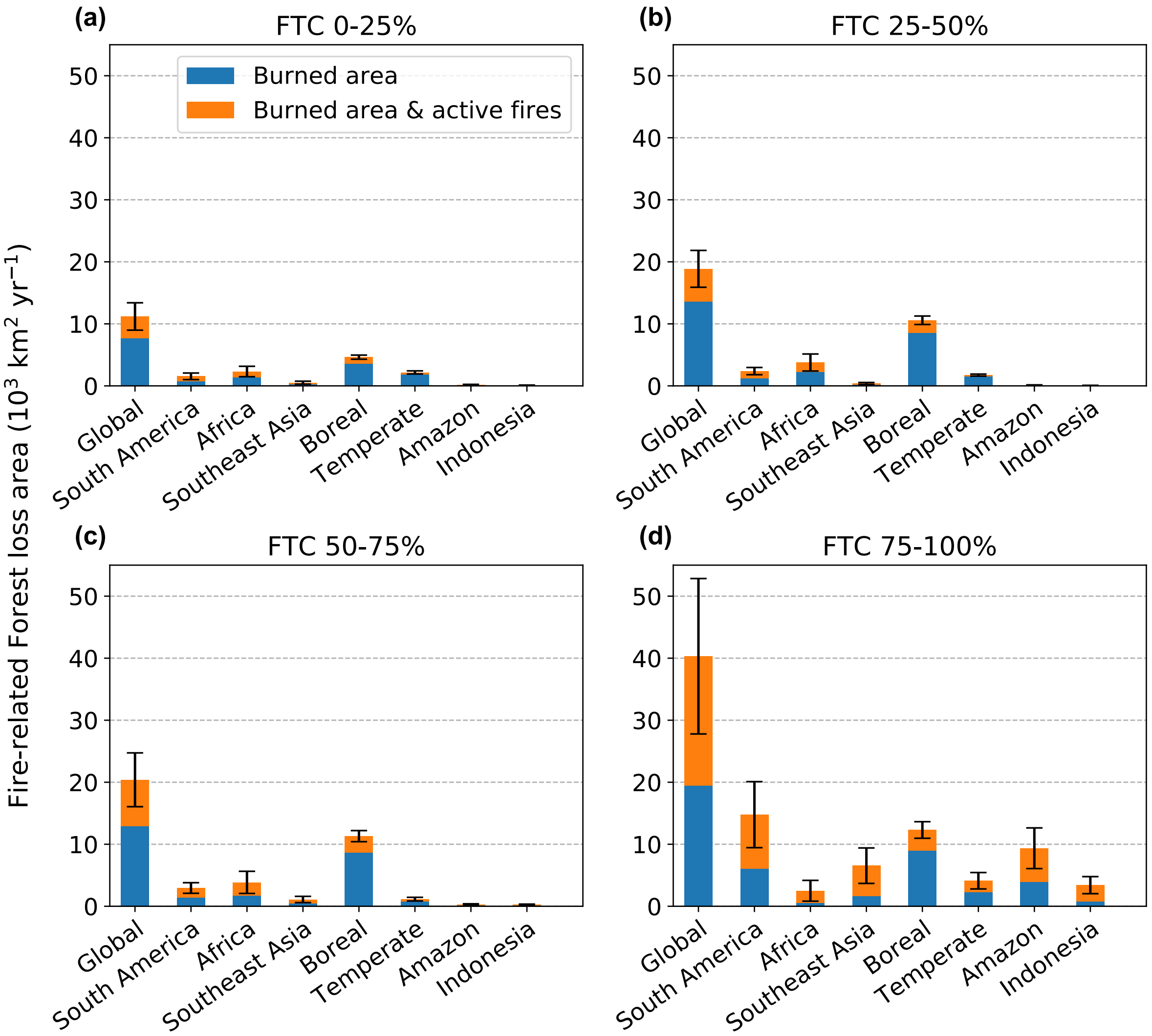

Figure S12: Comparison of fire-related forest loss estimates based on MODIS versus VIIRS active fires, in addition to MODIS burned area, showing **(a)** difference between minimum-estimates, **(b)** difference between best-estimates, and **(c)** scatter plot of the VIIRS versus MODIS best-estimate, displayed as 2d histogram. All values are based on 0.25° grid aggregates.

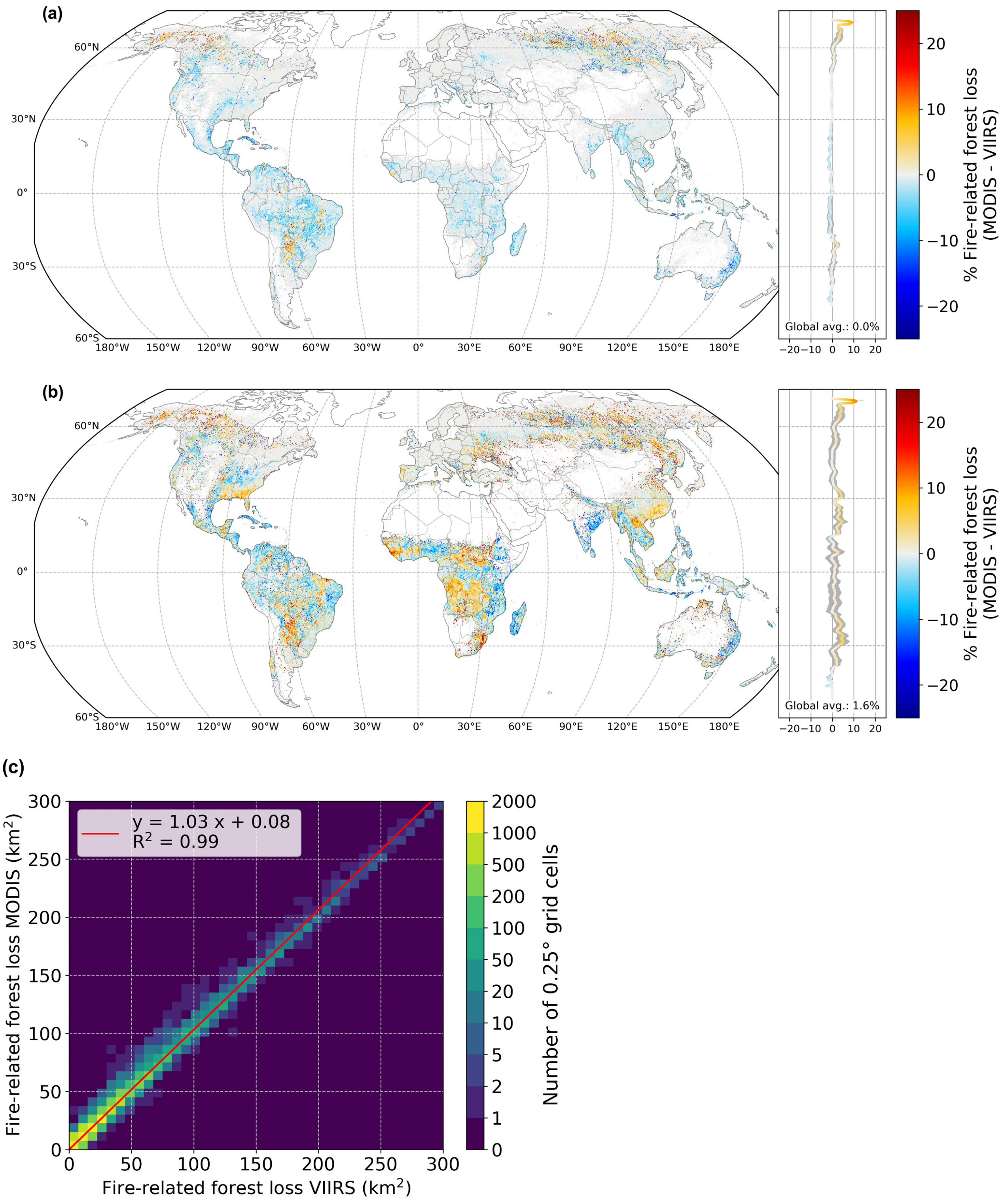

Figure S13: 2012-2018 average active fire pixel area per fire count, weighted by forest loss area per 500 m pixel, for **(a)** MODIS active fires, and **(b)** VIIRS active fires.

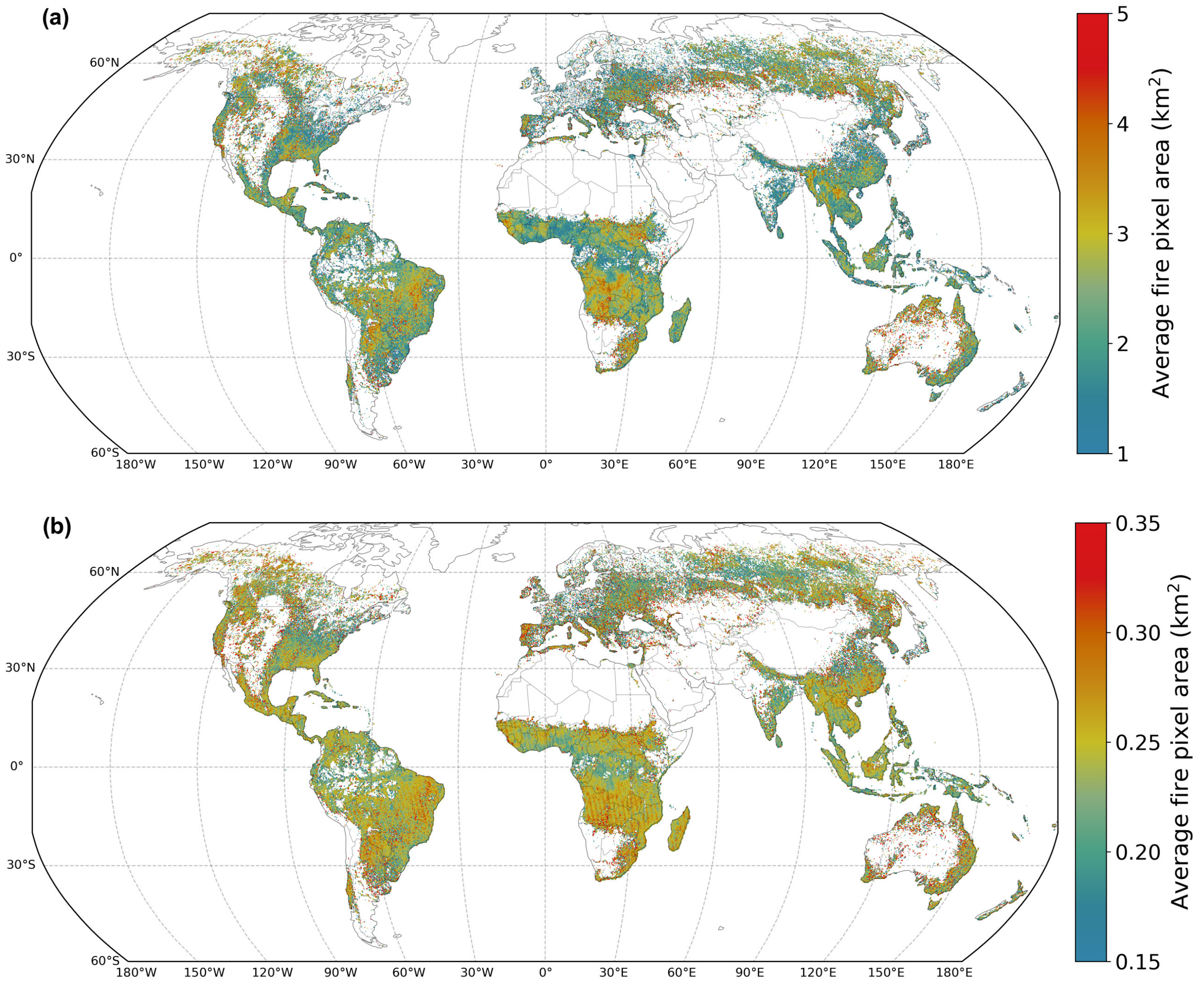

Figure S14: Comparison of fire-related forest loss derived from Sentinel-2 20 m burned area, and fire-related forest loss derived from MODIS 500 m burned area combined with MODIS or VIIRS active fires, for sub-Saharan Africa. Total forest loss represents all forest loss area in that year. Fire-related forest loss is shown per satellite detection product. Fire-related forest loss estimates based on MODIS and VIIRS active fires are additional to the part based on MODIS burned area, as according to our methodology.

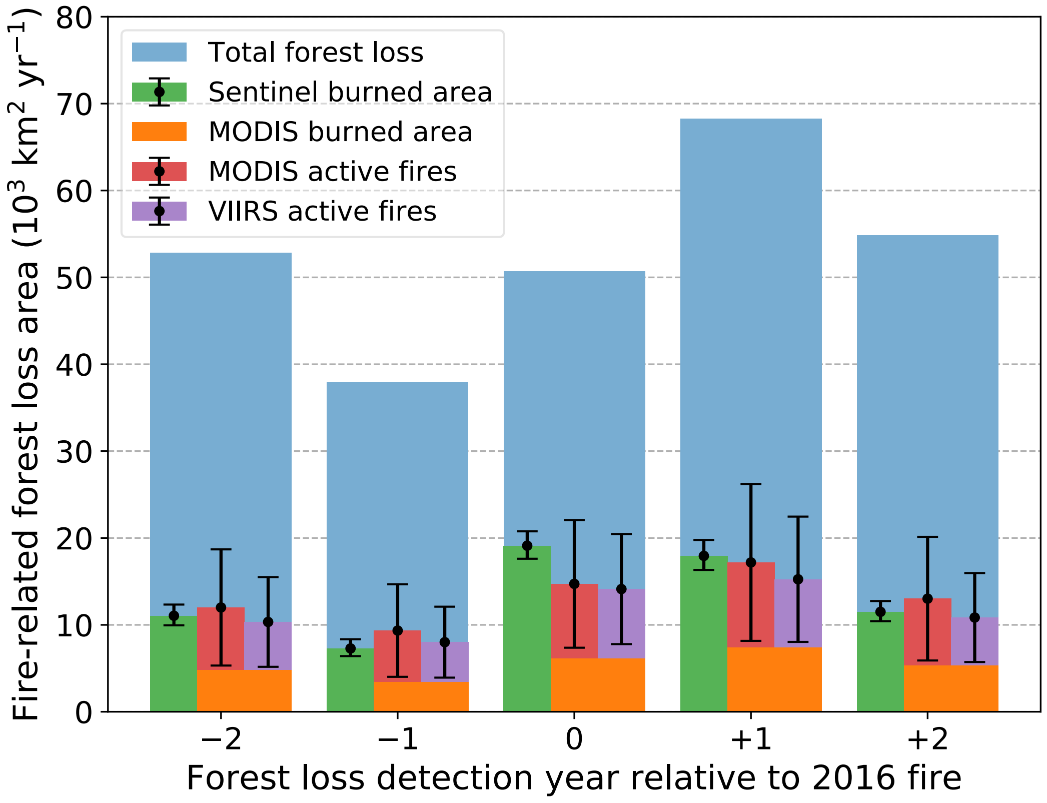

Figure S15: **(a)** Fraction of fire-related forest loss for 2016 (detection year 0 in Figure S14), based on Sentinel 20 m resolution burned area, and **(b)** and **(c)** the MODIS-based best and maximum estimates, respectively. Panels **(d)** and **(e)** show the difference between the Sentinel-based estimate versus the MODIS-based best and maximum estimates, respectively (i.e. panel **d** = **b** – **a** and panel **e** = **c** – **a**). Panels **(f)** and **(g)** scatter plots of the Sentinel-based estimate versus the MODIS-based best and maximum estimates, respectively, displayed as 2d histograms. All values are based on 0.25° grid aggregates. In panels **(a)** to **(e)** only 0.25° grid cells that contain a forest loss fraction of more than 0.1% are shown.

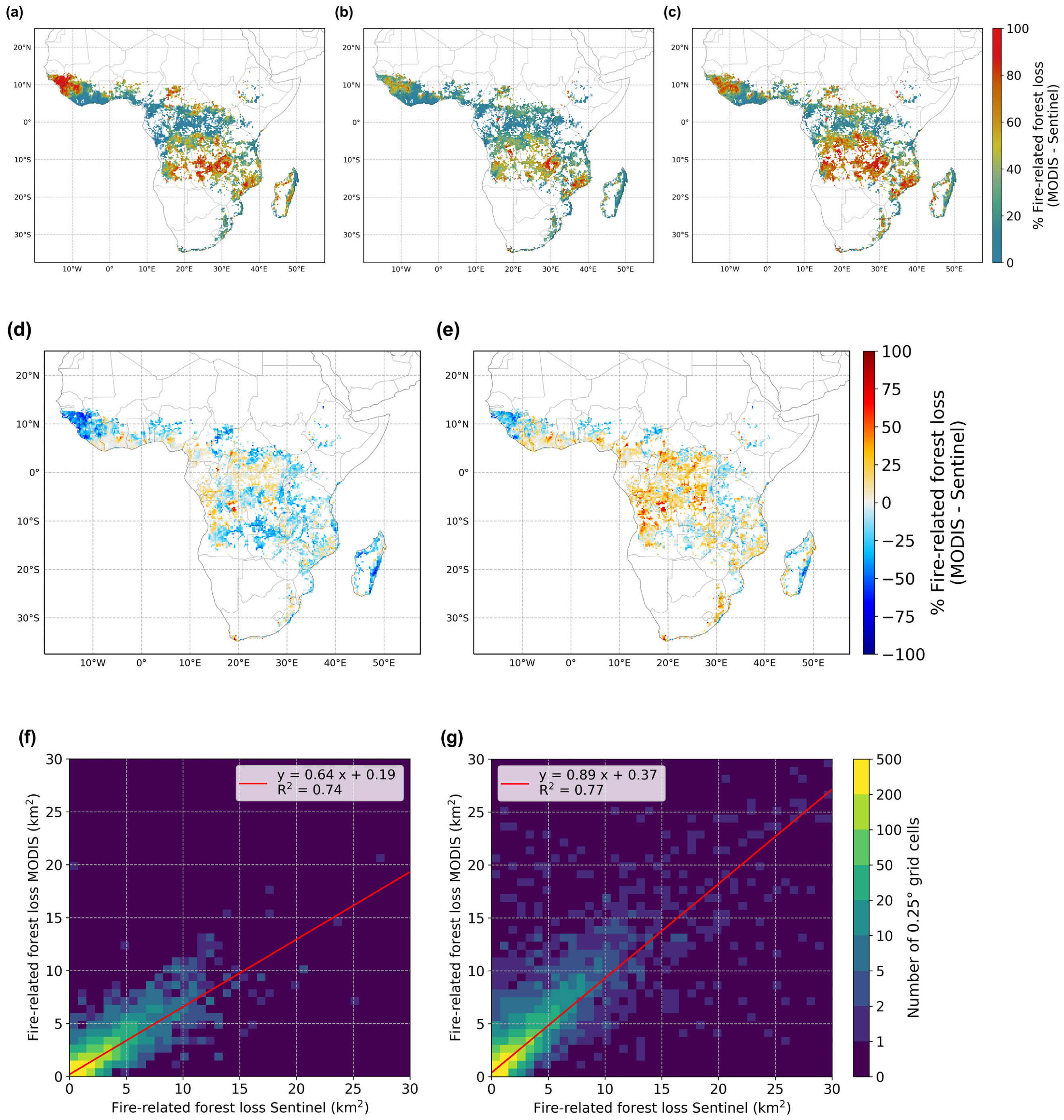

Figure S16: Lag analysis of monthly fire detections overlapping forest loss on 500 m pixel level, for fire detections from 24 months before until 24 months after the forest loss event. Histograms show monthly overlap of 2014-2016 forest loss with MODIS and VIIRS active fires, for the global average and the 14 GFED regions (see Figure 3 in van der Werf et al., 2017). Displayed fire-related forest loss based on active fire detections constitutes only active fire pixels outside of burned area, i.e. total fire-related forest loss minus the part by burned area). Monthly distributions per year were calculated by determining the spatial overlap of forest loss in year $t = 0$ (months 0 to 11) with monthly fires from years $t = -2$ to $t = +2$ (months -24 to 24) before and after the forest loss detection year. The resulting distributions for each forest loss year were then summed and divided by the number of years to create the annual average distributions as displayed.

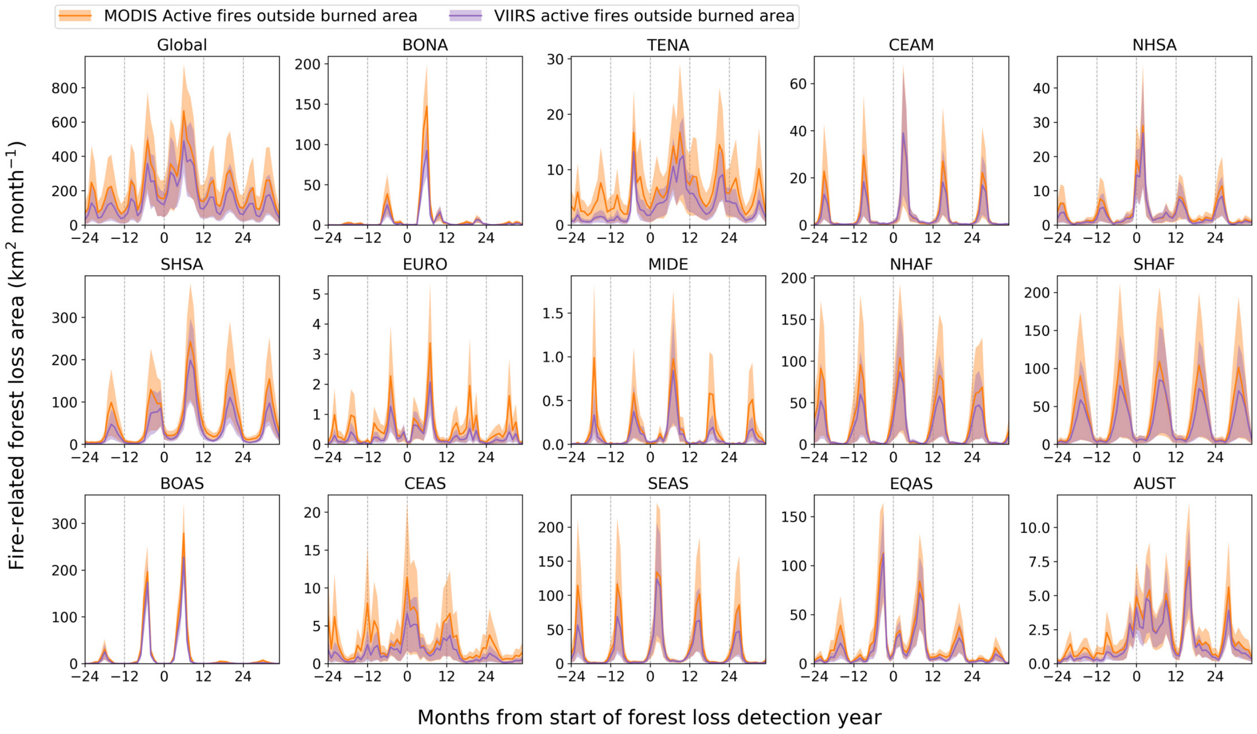

Supplement: Supplementary file 1 — Supplementary Material [file GCB-27-2377-s001.pdf]
